# The relationship between viscoelasticity and elasticity

**DOI:** 10.1098/rspa.2020.0419

**Published:** 2020-11-18

**Authors:** J. H. Snoeijer, A. Pandey, M. A. Herrada, J. Eggers

**Affiliations:** 1 Physics of Fluids Group, Faculty of Science and Technology, Mesa+ Institute, University of Twente, 7500 AE Enschede, The Netherlands; 2 Depto. de Mecánica de Fluidos e Ingeniería Aeroespacial, Universidad de Sevilla, 41092 Sevilla, Spain; 3 School of Mathematics, University of Bristol, Fry Building, Woodland Road, Bristol BS8 1UG, UK

**Keywords:** elasticity, viscoelasticity, capillarity, gels, instability

## Abstract

Soft materials that are subjected to large deformations exhibit an extremely rich phenomenology, with properties lying in between those of simple fluids and those of elastic solids. In the continuum description of these systems, one typically follows either the route of solid mechanics (Lagrangian description) or the route of fluid mechanics (Eulerian description). The purpose of this review is to highlight the relationship between the theories of viscoelasticity and of elasticity, and to leverage this connection in contemporary soft matter problems. We review the principles governing models for viscoelastic liquids, for example solutions of flexible polymers. Such materials are characterized by a relaxation time *λ*, over which stresses relax. We recall the kinematics and elastic response of large deformations, and show which polymer models do (and which do not) correspond to a nonlinear elastic solid in the limit *λ* → ∞. With this insight, we split the work done by elastic stresses into reversible and dissipative parts, and establish the general form of the conservation law for the total energy. The elastic correspondence can offer an insightful tool for a broad class of problems; as an illustration, we show how the presence or absence of an elastic limit determines the fate of an elastic thread during capillary instability.

## 1. Introduction

The aim of this review is to expose systematically the relationship between the theories of viscoelasticity and of elasticity, and to leverage what can be learned from this connection. Given the very mature state of these fields, there exist many excellent review articles and monographs that cover all aspects of elastic liquids and elastic solids in great detail [1–16]. With this review, we therefore do not attempt a broad overview of these research areas, but very specifically focus on how elasticity and viscoelasticity are related. This relationship is much more difficult to find in the literature, but it can greatly contribute to the understanding of contemporary developments involving soft materials at large deformations.

### (a) Soft materials and large deformations

Exceedingly soft solids, such as gels, elastomers and biological tissues, are extremely versatile and find numerous applications in nature and technology. Their mechanics is intricate: owing to their large deformability, soft solids can no longer be described within the framework of linear elasticity, but exhibit all the kinematic nonlinearities typical of the motion of fluids. This is the domain of large-deformation theory, which leads to nonlinear equations even if the elastic response of the material is perfectly linear.

**Figure 1.**
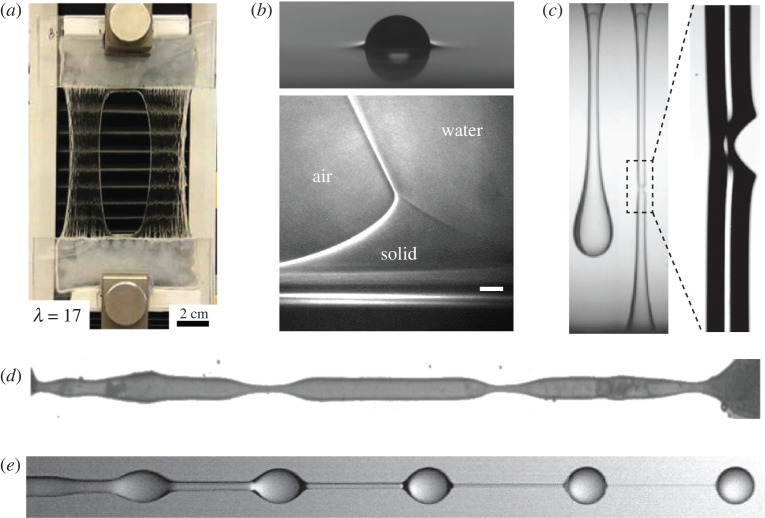
Soft matter at large stretch. (*a*) Tough solid: a sheet of a soft but very tough hydrogel that exhibits a reversible deformation when stretching up to a factor of 17. Even the presence of a hole in the centre did not nucleate any fracture. Adapted with permission from [17]. Copyright © Springer Nature. (*b*) Viscoelastic solid: liquid drops spreading over an elastomeric substrate cause large deformation at the contact line. The top panel reveals a ‘wetting ridge’ around a millimetric drop (courtesy Mathijs Van Gorcum). The bottom panel is a magnified view of the wetting ridge at three phase contact lines. The scale bar is 2 *μ*m. Adapted with permission from Park [18], under CC-BY 4.0 license. (*c*) Fracture in a viscoelastic liquid: the liquid is a functionalized micro-emulsion, forming transient networks, and exhibits brittle fracture. The thread radius at fracture is around 0.3 mm. Adapted with permission from [19]. Copyright © Royal Society of Chemistry. (*d*) Elastic thread: when sufficiently soft, a cylinder of cross-linked agar gel undergoes a Rayleigh–Plateau instability (cylinder radius 0.24 mm). Adapted with permission from [20]. Copyright © American Physical Society. (*e*) Viscoelastic thread: beads-on-a-string formation during the pinch-off of dilute (0.01 wt%) aqueous polyacrylamide solution undergoing capillary thinning (jet radius 0.3 mm). Adapted with permission from [21]. Copyright © Cambridge University Press.

Figure 1 provides various contemporary illustrations of soft matter at very large deformation. Figure 1*a* shows an extremely tough hydrogel [17]. It is specifically designed to reversibly resist very large stretches, up to a factor of approximately 20, without fracture. A second example, given in figure 1*b*, consists of a liquid drop on a solid polydimethylsiloxane substrate [18]. The liquid–vapour interface creates a sharp elastic deformation, in the shape of a ridge around the droplet’s edge [22–24]. Interestingly, the dynamical spreading of drops on elastomers is dramatically slowed down by this deformation: during spreading, the ridge is transported along with the droplet edge and induces very large dissipation inside the substrate [25–28]—without any irreversible damage to the material. Indeed, highly deformable solids often exhibit strongly viscoelastic behaviour, where the dissipation occurs during transients of deformation. Such dissipation is actually exploited in the design of pressure-sensitive adhesives [29–31], and can also, for example, explain the delayed snap-through instability of jumping toy poppers [32].

So both fluids and soft solids can exhibit dissipation, and both share the same nonlinear kinematics under large deformations. The fundamental distinction between a (hyper)elastic solid and a (Newtonian) fluid is that the former maintains a permanent memory of its initial or ‘reference’ state to which it relaxes, whereas in a simple fluid all configurations are equivalent and only rates of deformation are important. Accordingly, the natural description of a solid is a ‘Lagrangian’ viewpoint, which follows the path of each element of the continuum as labelled by the reference state, and all forces are determined from this mapping from the reference to the ‘current’ state. Fluid motion can also be described using Lagrangian paths, which is a natural point of view when considering mixing and advection problems [33,34], but has also proved to be a fruitful way of looking at classical fluid mechanics problems [35,36]. However, fundamentally Lagrangian trajectories are extremely intricate, even for very simple time-independent flows [33], and thus it is much simpler to disregard the history of each particle. Instead, in the Eulerian point of view one considers snapshots of the velocity field only, which is sufficient to calculate rates.

There are indeed numerous situations in which the material’s response exhibits both solid-like and fluid-like behaviour. Our focus is the connection that exists between fluid and solid mechanics in the limit of large relaxation times, and which persists in the case of large deformations. Here we remark that another relation exists that is different from the one that we address: it has been appreciated for a long time [37,38] that the linear equations for the velocity field of a viscous fluid, the so-called Stokes equations, and the equations of linear elasticity, in the incompressible limit of Poisson’s ratio being 1/2, are formally equivalent. For example, the theory of cracks in an elastic material [39] can be applied to the shape of free-surface cusps on the surface of a viscous fluid [40]. Taylor [41] noticed that the correspondence also applied to thin threads and sheets of viscous fluid, which on a short time scale are described by the nonlinear elastica equations and the equations for elastic sheets, respectively. This analogy has subsequently been derived more formally [42,43] and applied to many different physical situations.

As a particularly instructive example for the relationship between elasticity and viscoelasticity made in the limit of large relaxation times, we consider the capillary instability of cylindrical jets [20,44–46]. Figure 1*d* shows a cylinder of a fully cross-linked agar gel, which possesses a well-defined reference state [20]. Despite its elasticity, the cylinder exhibits a Rayleigh–Plateau instability that one usually associates with liquid jets [47]. The cross-linked network ultimately prohibits break-up and leads to the formation of thin elastic threads. For comparison, figure 1*e* shows a jet consisting of a dilute polymer suspension, a viscoelastic *liquid*; here break-up does occur, and the tenuous liquid filaments become thinner over time [47,48]. Conversely, complex liquids whose microstructure develops transient elastic networks can exhibit solid-like brittle fracture [19,49], as shown in figure 1*c*. In this case, the deformation is initially liquid-like but at some point breaks as if it were a solid.

It can be argued, as we will do, that viscoelastic liquids can be used as a universal modelling paradigm for a broad class of soft matter systems such as in figure 1. In contrast to elastic solids, viscoelastic liquids such as polymer solutions or emulsions do not possess a permanent reference state. Instead, they exhibit a fading memory of any prior deformation, which is characterized by a relaxation time *λ*, which is the time scale over which elastic stresses relax during flow. Such complex fluids are extremely common and important [2–4,50], as they occur whenever large and flexible molecules or other similar structural elements are present in the flow, as is the case in a vast range of biological and industrial contexts. In practice, soft materials with a complex internal structure possess a broad distribution of time scales, and can exhibit a power-law response [51,52] rather than the conventional exponential response.

Whenever a polymer is transported by a flow, it leaves its preferred reference state and exerts a force back on the liquid. To make the extremely complicated interactions between liquid and microstructure tractable, the polymer is often modelled as two beads, convected by the flow and connected by an elastic spring [2]. If the spring is soft, the polymer experiences large deformations, as the beads follow the complicated Lagrangian trajectories of the flow. As a result, the response becomes very nonlinear even if the spring is Hookean. A fluid in which the stress consists of a contribution of damped Hookean springs (damping due to friction with the solvent complemented by the Newtonian stress of the solvent) is known as an Oldroyd-B fluid. Owing to its conceptual simplicity it has become one of the most popular models of elastic liquids, although it neglects any nonlinear response of the spring as well as interactions between constituents.

The relaxation time *λ* of the model polymer derives from the ratio of the frictional force between a bead and the surrounding liquid divided by the spring constant, ensuring return to an equilibrium state. In weak flows, such that the relaxation time multiplied by a typical rate of deformation of the flow is small, the polymer remains close to its equilibrium shape, and only makes a linear, Newtonian contribution to the stress. Even if the flow is strong, on a time scale that is much larger than *λ*, the polymer will have ‘forgotten’ the deformations it experienced in the past. Only in the limit *λ* → ∞ will each bead follow its Lagrangian path as a passive tracer and produce an elastic response associated with large-deformation elasticity. In other words, upon varying the time scale *λ*, viscoelasticity continuously bridges the gap between a Newtonian liquid and a perfectly elastic solid. Various pioneering works are actually based on this idea, using a continuum formulation of viscoelasticity that is based on the theory of elasticity with an additional relaxation process [12,53–55].

**Figure 2.**
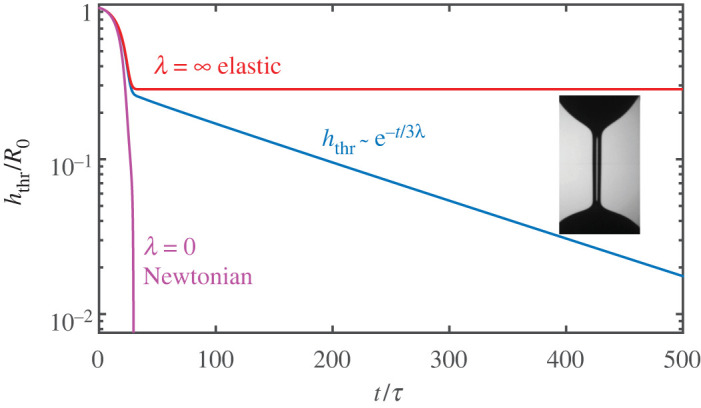
Thinning dynamics of a viscoelastic liquid thread. The inset shows a liquid thread between two drops of dilute polymer suspension (courtesy A. Deblais and D. Bonn; see also [56]). The main panel shows the thinning dynamics of the thread as described by an Oldroyd-B fluid, for different values of the relaxation time *λ*. The thread thickness *h*_thr_ is scaled by the initial jet radius *R*_0_, while time is scaled by the capillary time $\tau =\sqrt{\rho R_{0}^{3}/\gamma }$. The model continuously bridges between Newtonian liquids (*λ* = 0) and elastic solids (*λ* = ∞). (Online version in colour.)

We further illustrate the correspondence between elasticity and viscoelasticity using the thinning of a viscoelastic cylinder under capillary action (cf. figure 1*d*,*e*). Figure 2 shows the capillary thinning as modelled by the Oldroyd-B fluid. For *λ* = 0, the liquid has no memory and the thread thickness *h*_thr_ tends to zero like a power law [47,57], following a perfectly Newtonian pinch-off. For finite *λ*, polymers become increasingly stretched by the elongational flow near any potential pinch point, and the effective elongational viscosity increases exponentially. As a result, a very uniform thread is formed whose radius decreases exponentially on a time scale set by *λ*. At *λ* = ∞ one recovers a purely elastic behaviour, in this case that of a neo-Hookean solid. As the solid becomes increasingly deformed by surface tension, elastic stresses build up until they balance surface tension and a stationary thread of constant radius is formed.

### (b) This review

As highlighted above, this review focuses on the specific issue of how the theories of elasticity and of viscoelasticity are related, and to leverage what can be learned from this connection in the context of recent research. This explicit relation is not frequently explored, but, in fact, can be a very insightful and powerful tool for challenging problems, such as those in figure 1. As an example, we recently solved a long-standing problem in the break-up of dilute polymer suspensions by exploiting the elastic correspondence in the limit *λ* = ∞ [48]. Conversely, viscoelastic liquids probed at high rates have been used as a model soft elastic solid at large deformation [58].

The elastic correspondence offers a way to bridge the gap between different communities working in various areas of soft matter (biophysics, chemistry, engineering, fluid physics), which often use different modelling approaches to mechanics. These approaches are very well documented in established reviews and monographs [1–16]. However, there is a barrier to crossing the disciplines because of differences in mathematical formulation; the purpose of this review is to offer a unified exposition of elastic liquids and elastic solids. For example, while fluid mechanicians are mostly familiar with an Eulerian description, solid mechanics is most naturally expressed using the Lagrangian description. In addition, constitutive relations for liquids are usually formulated in terms of the stress tensor, while hyperelastic solids are defined by a (strain-dependent) free energy density [8]. We remark that a generalization of the concept of hyperelasticity is known as implicit elasticity [13,15], in which the elastic internal energy is dependent on both stress and strain, a situation we will not consider here.

We will use the elastic strain energy density of the polymers to present a systematic way of deriving an energy balance equation for viscoelastic fluids. While a mainstay of Newtonian fluid mechanics, energetic arguments are not much used for elastic fluids. Yet they reveal important characteristics of such fluids, as energy can now be stored temporarily in its elastic form, transported to other parts of the flow and eventually injected back as kinetic energy of the flow.

The review is organized as follows. In §2, we briefly summarize classical continuum theory, presenting side by side the formulations of viscoelasticity and of large-deformation elasticity. Section 3 explores what we call the ‘elastic correspondence’, by investigating viscoelastic liquid models in the limit *λ* → ∞. In particular, we show which models converge (or do not converge) to elastic solids when taking this limit. The elastic correspondence is then exploited in detail by the example of capillary thinning in §4, highlighting the importance of whether or not the elastic correspondence exists. This also offers a new modelling paradigm to viscoelastic solids, based on models of viscoelastic liquids. Section 5 discusses a thermodynamic approach to viscoelastic liquids, and we discuss the relation between stress, energy and dissipation. We close with a discussion in §6.

## 2. Classical continuum theory

### (a) Viscoelastic fluids

The equations of motion for viscoelastic fluids are most commonly expressed in the Eulerian description, using a velocity field **v**(**x**, *t*) that is a function of space **x** and time *t*. Here we consider the fluid to be incompressible, ∇⋅v=0, and described by the momentum balance

2.1 $$\rho \left(\frac{\partial \text{v}}{\partial t}+\text{v}\cdot \nabla \text{v}\right)=\nabla \cdot \sigma ,$$

where **σ** is the stress tensor. The stress tensor is split into a Newtonian contribution (coming, for example, from a solvent) and a polymeric (viscoelastic) contribution **σ**_*p*_,

2.2 $$\sigma =-p\text{I}+\eta_{s}\dot{\gamma }+\sigma_{p}\equiv -p\text{I}+\tau ,$$

where the deviatoric stress **τ** is the contribution excluding the pressure. We defined the rate-of-deformation tensor to be

2.3 $$\dot{\gamma }=(\nabla \text{v})+{\left(\nabla \text{v}\right)}^{\text{T}}$$

and *η*_*s*_ is the solvent viscosity. Any isotropic contribution to the stress can be written as part of the pressure *p*. The non-Newtonian contribution **σ**_*p*_ originates from the presence of the microstructure inside the fluid. Though we will refer to **σ**_*p*_ as the ‘polymeric stress’, having in mind dilute polymer suspensions, the concept equally applies to emulsions whose microstructure is described by droplet deformations [59,60]. The stress **σ**_*p*_ is governed by a separate evolution equation, the constitutive equation, which encodes the non-Newtonian properties of the fluid. Specifically, for viscoelastic liquids, the constitutive equation describes the relaxation of stress over a time scale *λ*.

Many different approaches to modelling **σ**_*p*_ can be found in the literature [2–7,9,50]; these can be roughly divided into two categories. The first approach is that the polymer stress **σ**_*p*_ should be a functional of the *history of deformation* [5,9]. These constitutive relations are expressed either as a differential equation or in an integral form. In the latter case, one explicitly performs an average over past deformations with a weight factor that encodes the fading memory. Many of these constitutive equations are based on systematic expansions, where at each order one gradually adds more information of the deformation history and/or more nonlinearity [5]. The advantage of such an approach is that the calculation of stress is direct, though care must be taken that the formulated model is thermodynamically consistent [6].

In the second approach, the polymer stress is not expressed directly in terms of the deformation, but rather in terms of an *order parameter* field, **A**(**x**, *t*), that characterizes the state of the polymer (or, more generally, of the microstructure). In this approach, the polymeric stress is written as **σ**_*p*_ = **σ**_*p*_(**A**). The coupling to the deformation history is then achieved by a separate relaxation equation for **A**(**x**, *t*), which describes how the microstructure evolves over time. The advantage of an order parameter description is that it can be embedded in a thermodynamically consistent framework, as done, for example, in the bracket [6] and the GENERIC [7] formalisms. For polymer solutions and emulsions, the natural order parameter **A** is the so-called *conformation tensor*: it is a second-rank tensor that characterizes the amount of stretch of the polymer. The tensorial nature arises since stretching will in general be different along different directions. It has the property that the polymer stress vanishes when **A** = **I**, where **I** is the identity tensor, and its principal values represent the stretches along principal directions. Another merit of the conformation tensor description is that it can be derived by coarse-graining microscopic models based on suspended bead-and-spring dumbbells [2].

As we will see below, the formulation in terms of a conformation tensor offers a natural connection to the theory of elasticity—which is the central purpose of this review. Therefore, in the remainder of the paper we focus primarily on constitutive modelling based on a conformation tensor. In §3e, and in a few other places, we briefly discuss how the conformation tensor models are connected to descriptions based on the history of deformation. Though the relation **σ**_*p*_(**A**) and the evolution equation for **A** can often be derived from microscopic models, we here follow a purely continuum approach, without referring to any microscopic model. Once again, the continuum description has the advantage of exposing the correspondence to the theory of elasticity—the ‘elastic limit’ is obtained by considering viscoelastic fluid models in the limit *λ* → ∞. We will see that this elastic correspondence completely determines the dependence **σ**_*p*_(**A**).

#### (i) The subtlety of the time derivative

Let us start by considering the simplest type of model based on the conformation tensor. In this case, the relation **σ**_*p*_(**A**) is linear

2.4 $$\sigma_{p}=\mu (\text{A}-\text{I}),$$

where *μ* is the analogue of the elastic shear modulus. The state variable **A** must have the property that it evolves towards its relaxed state **A** = **I** in the limit of long times. Once more, the simplest way of doing this is through a linear relaxation law

$$\dot{\text{A}}=-\frac{1}{\lambda }(\text{A}-\text{I}),$$

which is known as a Maxwell model. In such a model, an initial condition **A** relaxes exponentially towards **A** = **I** on a time scale *λ*. However, it has been known for a long time [2,61] that, for the dynamics of a second-rank tensor to be *frame invariant* [50], i.e. to be independent of the frame of reference, the ordinary time derivative needs to be replaced by one of two frame-invariant derivatives or a linear combination of the two. The so-called *upper convected derivative*

2.5 $$\overset{▽}{\text{A}}=\frac{\partial \text{A}}{\partial t}+\text{v}\cdot \nabla \text{A}-{\left(\nabla \text{v}\right)}^{\text{T}}\cdot \text{A}-\text{A}\cdot (\nabla \text{v})$$

is derived from the requirement that its components transform consistently as the components of a contravariant tensor. The first two terms on the right are the convected derivative of a material point, ensuring Galilean invariance; the last two terms make sure that **A** transforms correctly under deformations by the flow. However, a covariant formulation does equally well from the point of view of frame invariance, but yields a different derivative, known as the *lower convected derivative*,

2.6 $$\overset{△}{\text{A}}=\frac{\partial \text{A}}{\partial t}+\text{v}\cdot \nabla \text{A}+\text{A}\cdot {\left(\nabla \text{v}\right)}^{\text{T}}+(\nabla \text{v})\cdot \text{A}.$$

As the names suggest, these derivatives have natural geometric interpretation in curvilinear coordinates that are convected with the flow [50,61,62]. For completeness, the curvilinear description is given in appendix A, where we discuss in detail the geometric interpretation.

From the point of view of frame invariance, one is thus left with a somewhat unpleasant ambiguity. Namely, the derivatives $\overset{▽}{\text{A}}$ and $\overset{△}{\text{A}}$, and linear combinations of the two, are equally admissible when building a theory for viscoelastic fluids. The resulting mechanical behaviour, however, is manifestly different depending on the choice of the derivative. In particular, when using the upper convected derivative, the stress will grow exponentially in a strong extensional flow, as expected from a bead-and-spring model [2], where the two beads will be separated by the flow. However, the ambiguity of the time derivative can be lifted more generally, without relying on any microscopic model. This becomes particularly clear when working out the correspondence to the theory of elasticity.

Using the upper convected derivative, a linear relaxation law thus takes the form

2.7 $$\overset{▽}{\text{A}}=-\frac{1}{\lambda }(\text{A}-\text{I}).$$

It is instructive to combine (2.4) and (2.7), such that we have a single equation of motion for the polymeric stress. Introducing the polymeric viscocity *η*_*p*_ = *μλ*, this gives

2.8 $$\sigma_{p}+\lambda {\overset{▽}{\sigma }}_{p}=\eta_{p}\dot{\gamma }.$$

**Figure 3.**
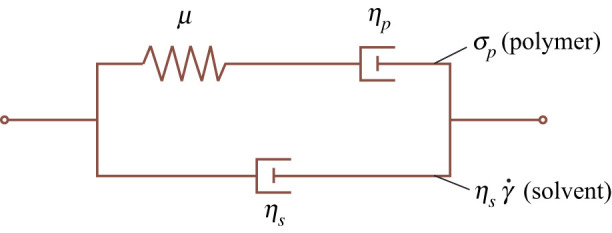
Qualitative representation of the Oldroyd-B fluid. The deviatoric stress is given by the sum of the Newtonian stress of the solvent (viscosity *η*_*s*_) and the viscoelastic stress of the upper convected Maxwell fluid. The latter is characterized by a polymer viscosity *η*_*p*_ and an elastic modulus *μ*. The ratio of *η*_*p*_/*μ* gives the polymer relaxation time *λ*. Note that the spring-dashpot analogy is not to be taken quantitatively, certainly not at large deformations where one encounters strongly nonlinear responses.

This is a tensorial form of the upper convected Maxwell model. The stress tensor (2.2) with **σ**_*p*_ given by (2.8) is known as the Oldroyd-B model [2]; in the limit of vanishing rates of deformation, it describes a Newtonian fluid of total viscosity *η*_0_ = *η*_*s*_ + *η*_*p*_. Figure 3 provides a schematic of the Oldroyd-B fluid, whose mechanical response consists of a Newtonian solvent in parallel with a so-called upper convected Maxwell fluid. When omitting the solvent, one recovers the upper convected Maxwell model.

Equation (2.8) is an example of a constitutive relation where **σ**_*p*_ is expressed in terms of the history of deformation. Here this is in the form of a differential equation, where deformation is encoded in $\dot{\gamma }$ and in ∇v in the upper convected derivative. In fact, (2.8) is a special case of the more general expansion by Oldroyd [5], known as the eight-constant model, in which all terms to second order in stress and strain rate are retained, while respecting frame invariance. We return to Oldroyd’s eight-constant model in §3e. For now, let us remark that, in such an expansion, there is no mathematical ground to anticipate whether taking ${\overset{▽}{\sigma }}_{p}$ or ${\overset{△}{\sigma }}_{p}$ in (2.8) would do a better job in describing polymeric fluids.

Although the Oldroyd-B model is very popular owing to its simplicity, there are many relevant physical effects which are not captured. For example, it incorrectly describes the shear-thinning behaviour of polymeric fluids, and in a strong extensional flow the stress will grow indefinitely. This can be avoided by incorporating the fact that the spring can only reach a finite extension, by making the spring constant increase as full extension is reached. There exist numerous extensions of the Oldroyd-B equations in that spirit; for example, taking into account nonlinearity in both (2.4) and (2.7), or in the solvent contribution in (2.2). In §5c, we supply a list of various models. Apart from the question of frame invariance, models have to be consistent with the requirements of thermodynamics [6,7,59,60,63].

### (b) Elasticity

We now turn to a brief exposition of the theory of elasticity [8,64]. While fluid mechanics is usually expressed in the Eulerian formulation, using the spatial coordinates **x** to describe the system, nonlinear (finite deformation) elasticity is written in a Lagrangian formulation based on material coordinates. This is because elastic solids exhibit a well-defined (undeformed) reference state, in which elastic energy is minimal and the elastic stress vanishes. The material coordinates in this reference state are denoted by **X**. Deformations are described by a mapping **x** = *χ*(**X**, *t*), where **x** denotes the position of a material point after the deformation, which used to be at **X** before the deformation. In fluid mechanics, the mapping is known as a Lagrangian path of a particle with label **X**. Flow corresponds to the case where the mapping *χ* is time dependent, though even static deformations are of interest in the context of solid mechanics.

The theory of (hyper)elastic solids is based on the idea that deformations are perfectly reversible, without any dissipation, so that their constitutive behaviour can be formulated in terms of an elastic free energy. Figure 1*a* offers a spectacular example of a solid with a perfectly reversible response. If the medium is isotropic, the density of elastic energy *W* can only depend on the change in distance between material points generated by a deformation [10]. To evaluate this change of distance, we introduce the deformation gradient tensor **F** = ∂**x**/∂**X**. Namely, if d*s* is the distance between two points which used to be a distance d*S* apart, we obtain [8], using d**x** = **F** · d**X**, that

2.9 $$\text{d}s^{2}-\text{d}S^{2}=\text{d}{\text{X}}^{\text{T}}\cdot ({\text{F}}^{\text{T}}\cdot \text{F}-\text{I})\cdot \text{d}\text{X}.$$

The deformation is thus encoded in Green’s deformation tensor **C** = **F**^T^ · **F**, which is a symmetric second-rank tensor that is defined on the reference configuration (the tensor (**F**^T^ · **F** − **I**)/2 is called the finite-strain tensor). The stored density of elastic energy must be a function of **C**, or, more specifically, of the invariants of **C**. The energy function *W* = *W*(**C**) must also have the property that it assumes a minimum for **C** = **I**, when the material is undeformed from its reference state. This means that any deformation costs energy, which is a necessary condition for the unstressed state to be stable.

Given that we are interested in the connection to Eulerian theory for viscoelastic liquids, we will not pursue further the Lagrangian formulation of elasticity—for that we refer to [8,64]. All we need for the present discussion is that **C** shares the same eigenvalues as those of the Finger tensor [8], defined as **B** = **F** · **F**^T^. This can be understood when expressing **F** along principal directions. In that case **F** is diagonal and the eigenvalues {Λ_*i*_} are the principal stretches, expressing the corresponding ratio of d*s* and d*S*; the corresponding **B** and **C** are then also diagonal, with identical components $\left\{\Lambda_{i}^{2}\right\}$. So, why do we wish to use the Finger tensor? In contrast to **C**, the Finger tensor **B** is an Eulerian tensor, defined on the current configuration,^1^ and is therefore more appropriate when connecting to the Eulerian description of viscoelastic liquids. Given that the invariants of **C** are the same as those of **B**, we can thus write *W* = *W*(**B**) for the elastic free energy density.

Though not necessary, from now we on focus on incompressible deformations (as is the case for most polymeric solids), for which det(F)=1. Once the free energy is specified, the (Cauchy) stress tensor for incompressible media follows as [8,64]

2.10 $$\sigma_{p}=\frac{\partial W}{\partial \text{F}}\cdot {\text{F}}^{\text{T}}=2\frac{\partial W}{\partial \text{B}}\cdot \text{B},$$

where in the second step we exploited the symmetry of **B**. This expression is a consequence of the virtual work principle [9], which requires that any change in the elastic energy density satisfies [10]

2.11 $$\frac{\text{d}W}{\text{d}t}=\sigma_{p}:{\left(\nabla \text{v}\right)}^{\text{T}}=\frac{1}{2}\sigma_{p}:\dot{\gamma },$$

where in the second step we used the symmetry of **σ**_*p*_. The derivation of (2.10) and (2.11) will be spelled out in §5. As we argued before, *W* can only be a function of one of the invariants of **B**, which can be written as

2.12 $$I_{1}=B_{kk},\;I_{2}=\frac{1}{2}(B_{kk}^{2}-B_{ij}B_{ij})\;\text{and}\;I_{3}=\det(\text{B}),$$

where we recall that $I_{3}=\det{\left(\text{F}\right)}^{2}=1$ for incompressible media. Hence, we can write the free energy as a function of the first two invariants only: *W*(*I*_1_, *I*_2_). The constraint *I*_3_ = 1 will be ensured by an isotropic pressure acting as a Lagrange multiplier. Using the relation between energy and stress (2.10), and the definitions of the invariants (2.12), we obtain

2.13 $$\sigma_{p}=2W_{1}\text{B}+2W_{2}(\text{tr}(\text{B})\text{B}-\text{B}\cdot \text{B}),$$

where *W*_1_ ≡ ∂*W*/∂*I*_1_ and *W*_2_ ≡ ∂*W*/∂*I*_2_. This can be simplified using the Cayley–Hamilton theorem, which reads

2.14 $$\det(\text{B}){\text{B}}^{-1}={\text{B}}^{2}-\text{tr}(\text{B})\text{B}+\frac{1}{2}(\text{tr}{\left(\text{B}\right)}^{2}-\text{tr}({\text{B}}^{2}))\text{I}.$$

Using $\det(\text{B})=\det{\left(\text{F}\right)}^{2}=1$, the stress can now be written as

2.15 $$\sigma_{p}=2W_{1}(\text{B}-\text{I})+2W_{2}(\text{I}-{\text{B}}^{-1}),$$

where for convenience we have absorbed an isotropic contribution into the pressure.

The derivatives *W*_1_ and *W*_2_ can be arbitrary nonlinear functions of the invariants *I*_1_ and *I*_2_. If *W*_1_ = *μ*/2 and *W*_2_ = 0, one finds the neo-Hookean model. The corresponding neo-Hookean energy reads *W* = (1/2)*μ*(*I*_1_ − 3), while using (2.15) one finds the stress to be **σ**_*p*_ = *μ*(**B** − **I**). Elastic models that contain both *W*_1_ and *W*_2_ as constants, so that the energy density is a linear combination of *I*_1_ and *I*_2_, go by the name of Mooney–Rivlin solids.

Interestingly, the neo-Hookean stress **σ**_*p*_ = *μ*(**B** − **I**) is of the same form as the viscoelastic expression for stress obtained in (2.4), for the Oldroyd-B fluid. The connection follows upon replacing the Finger tensor **B** by the conformation tensor **A**. This correspondence is not a coincidence. According to (2.9), the Finger tensor **B** measures the amount of stretching due to deformation of the entire medium. Similarly, we had postulated that the conformation tensor **A** provides a measurement of the amount of stretch—albeit not of the entire medium, but only of the polymer inside the solvent. Let us now proceed towards making this correspondence more rigorous.

### (c) Kinematics: the Eulerian–Lagrangian connection

To make a connection between Eulerian models for polymeric liquids and the Lagrangian formulation of elasticity, we need to find out what are the deformations generated through transport by the velocity field **v**. The velocity field is connected to the motion of material points by **v** = d**x**/d*t*, where d/d*t* is a time derivative at constant material point **X**. Then it follows from the chain rule that [3,50,65]

2.16 $$\frac{\text{d}\text{F}}{\text{d}t}={\left(\nabla \text{v}\right)}^{\text{T}}\cdot \text{F}\;\text{and}\;\frac{\text{d}{\text{F}}^{-1}}{\text{d}t}=-{\text{F}}^{-1}\cdot {\left(\nabla \text{v}\right)}^{\text{T}},$$

where ${\left(\nabla \text{v}\right)}_{ij}=\partial_{i}v_{j}$. The relation (2.16) permits us to calculate the deformation gradient tensor from **v**, and thus to pass from an Eulerian to a Lagrangian description.

Many kinematic relations can now be derived, but here we immediately turn to the main point of interest: the convected time derivatives of an Eulerian tensor **A**(**x**, *t*), and its relation to the Lagrangian mapping **F**(**X**, *t*), which is central to elasticity theory. To achieve that, we use the following identity relating the upper convective derivative and the time derivatives of the mapping [50]:

2.17 $$\overset{▽}{\text{A}}=\text{F}\cdot \left[\frac{\text{d}}{\text{d}t}({\text{F}}^{-1}\cdot \text{A}\cdot {\text{F}}^{-T})\right]\cdot {\text{F}}^{\text{T}}.$$

Making use of (2.16), the explicit evaluation of the time derivative in (2.17) indeed gives the original definition (2.5) of the upper convected derivative. The representation (2.17) of the upper convected derivative has a natural interpretation. Since convection plays no role in the domain of material coordinates, one first projects the Eulerian tensor **A** back to the Lagrangian domain, using the inverse transformation ${\text{F}}^{-1}\cdot \text{A}\cdot {\text{F}}^{-T}$. Then the time derivative is performed on the Lagrangian domain without suffering from the effect of flow. Finally, the result is returned to the Eulerian domain to yield a truly objective tensorial time derivative. Thus, we see that the tensor **F** plays a curious double role. On the one hand, as seen from (2.9), it produces a measure of elastic deformation, as defined by Green’s deformation tensor. On the other hand, as implied by (2.17), it is also a ‘machine’ which transforms between reference and current state. This is because **F** is a two-point tensor with one leg on the reference configuration and the other on the current configuration.

However, the above procedure is not unique. Namely, instead of ${\text{F}}^{-1}\cdot \text{A}\cdot {\text{F}}^{-T}$ one can also construct a Lagrangian tensor as **F**^T^ · **A** · **F**. Following the same procedure as above, this gives an alternative time derivative

2.18 $$\overset{△}{\text{A}}={\text{F}}^{-T}\cdot \left[\frac{\text{d}}{\text{d}t}({\text{F}}^{\text{T}}\cdot \text{A}\cdot \text{F})\right]\cdot {\text{F}}^{-1},$$

which again agrees with the lower convected derivative as defined in (2.6).

So how can one decide which one of the two transformations (or a mixture of both) is appropriate? The answer is that this depends on the physical meaning of **A**. In the curvilinear description, developed in appendix A, it is shown that the two transformations, ${\text{F}}^{-1}\cdot \text{A}\cdot {\text{F}}^{-T}$ and **F**^T^ · **A** · **F**, give the transformations of the contravariant and covariant components of the tensor **A**, respectively. In the curvilinear framework, the relaxation equation for the conformation tensor—which measures the stretching of the polymer—is naturally expressed in contravariant form.^2^ Hence, the upper convected derivative emerges, consistently with the results of the bead-and-spring model. Rather than following the curvilinear description, we now proceed by a more intuitive discussion of this result through the elastic limit *λ* → ∞ of viscoelastic models.

## 3. The limit *λ* → ∞: do all viscoelastic models converge to elastic solids?

The central purpose of the paper is to lay out the relationship between viscoelastic models and the theory of elasticity. It is clear that this connection is to be found by investigating the limit of infinite relaxation time, for which we expect a perfect memory of any preceding deformation. Therefore, the precise question we wish to address is whether a given viscoelastic fluid model, in the limit *λ* → ∞, converges to the constitutive relation of an elastic solid. The latter is defined by (2.15) for an incompressible elastic solid. It will turn out that this elastic correspondence exists only for a specific class of rheological models. With this perspective, we will revisit the so-called Pipkin diagram that is used classically to summarize the regimes of viscoelastic responses, and comment on the meaning of the elastic correspondence for viscoelastic solids.

### (a) An example: affine motion

#### (i) The conformation tensor in the elastic limit

We start by considering rheological models that involve the upper convected derivative of the conformation tensor, an example of which is given by the Oldroyd-B fluid (2.7). In the elastic limit, *λ* → ∞, this class of models reduces to

3.1 $$\overset{▽}{\text{A}}=0.$$

This equation describes the evolution of the conformation tensor in the elastic limit, induced by a flow **v**(**x**, *t*).

Upon inspection of (2.17), one easily verifies that the Finger tensor **B** = **F** · **F**^T^ has the property that $\overset{▽}{\text{B}}=0$ [3]. Hence, we have found a perfectly valid solution to (3.1), namely **A** = **B**. For the Oldroyd-B fluid, where we had **σ**_*p*_ = *μ*(**A** − **I**), we thus find that the polymer stress in the elastic limit exactly reduces to that of a neo-Hookean solid, **σ**_*p*_ = *μ*(**B** − **I**). When omitting the solvent viscosity in the Oldroyd-B fluid, the model reduces to the upper convected Maxwell fluid. The above analysis thus demonstrates that, in the limit *λ* → ∞, the upper convected Maxwell fluid is strictly identical to a neo-Hookean solid. In some cases the formulation of fluid models was in fact based on the observation that the Finger tensor vanishes [54], allowing a natural connection between viscoelasticity and elasticity.

It is clear that this route provides the correspondence between viscoelasticity and elasticity we were looking for. Equation (3.1) applies not only to the Oldroyd-B fluid, but also to any model for which the relaxation is of the form $\lambda \overset{▽}{\text{A}}=f(\text{A})$. It is therefore instructive to integrate (3.1) more formally, and find the general solution. This can be done by multiplying (2.17) by **F**^−1^ from the left and ${\text{F}}^{-T}$ from the right, which enables us to integrate in time to obtain ${\text{F}}^{-1}\cdot \text{A}\cdot {\text{F}}^{-T}\equiv {\text{D}}_{0}=\text{const}$. Correspondingly, we find

3.2 $$\text{A}=\text{F}\cdot {\text{D}}_{0}\cdot {\text{F}}^{\text{T}}.$$

Here **D**_0_ is a constant (time-independent) Lagrangian tensor, defined on the reference domain, which is therefore independent of the mapping; **D**_0_ can be viewed as an integration constant and can be determined from initial conditions. To illustrate this, we consider a case where there is a pre-stress $\sigma_{p}^{\left(0\right)}$ in the initial state for which **F** = **I**. For the case of a simple neo-Hookean relation (2.4), it follows that

3.3 $$\text{A}=\text{F}\cdot {\text{F}}^{\text{T}}+\frac{1}{\mu }\text{F}\cdot \sigma_{p}^{\left(0\right)}\cdot {\text{F}}^{\text{T}}.$$

In the particular case of a stress-free initial condition, we recover **A** = **B**, for which the reference state coincides with the initial condition. This illustrates how the concept of a reference state, central in the theory of elasticity, emerges in viscoelastic liquids as *λ* → ∞: it appears as an integration constant that can be determined from the initial condition.

#### (ii) Kinematic interpretation

We are now in a position to give a kinematic interpretation to the relaxation equation $\lambda \overset{▽}{\text{A}}=f(\text{A})$, making use of the elastic limit *λ* → ∞. In this limit, we have seen that the upper convected derivative implies that the conformation tensor **A** (stretching of the polymer) evolves in the exact same way as the Finger tensor **B** (stretching by the flow **F**). Hence, the polymer stretches simply by following the flow. This type of evolution of the conformation tensor due to deformation is called *affine*, in the sense that it follows the flow perfectly. The concept of affine motion, in conjunction with the upper convected derivative, appears naturally in microscopic bead–spring models. There, the upper convected derivative appears when the vector describing the orientation and length of the spring is transported in the same way as any vector moving along with the fluid [3]. However, such kinematic considerations do not require any specific microscopic model and can be inferred from purely continuum considerations (the general case for finite *λ* will be discussed in §5).

If on the other hand the relaxation law is based on the lower convected derivative, such that *λ* → ∞ implies $\overset{△}{\text{A}}=0$, using (2.18) we find $\text{A}={\text{B}}^{-1}\equiv {\text{F}}^{-T}\cdot {\text{F}}^{-1}$. This corresponds to a response in a direction opposite the flow. Hence, if we had elected a conformation tensor that measures the *inverse* of the polymer stretching, affine motion requires the use of the lower convected derivative. Let us illustrate these two cases using the simple elongational flow

3.4 $$v_{r}=-\frac{1}{2}\dot{\epsilon }r\;\text{and}\;v_{z}=\dot{\epsilon }z.$$

Integrating (2.16) with initial condition **F** = **I** one obtains

3.5 $$\text{B}=\left(\begin{array}{ccc} {\text{e}}^{-\dot{\epsilon }t} & 0\\ 0 & {\text{e}}^{2\dot{\epsilon }t} & \end{array}\right)\;\text{and}\;{\text{B}}^{-1}=\left(\begin{array}{ccc} {\text{e}}^{\dot{\epsilon }t} & 0\\ 0 & {\text{e}}^{-2\dot{\epsilon }t} & \end{array}\right).$$

In other words, **B** describes stretching in the *z*-direction and contraction in the radial direction that is generated by the flow **v**, while **B**^−1^ describes the inverse. More precisely, the eigenvalues of **B**^−1^ represent the ratio of the change in surface areas normal to the stretching direction [3].

The important conclusion here is that the choice of the conformation tensor as a measure of the polymer stretch (or its inverse) singles out the use of the upper (lower) convected derivative as the natural operator to specify affine transport induced by the flow. Since for polymers it is common that **A** measures the stretch, the affine transport implies the upper convected derivative.

### (b) A counterexample: non-affine motion

By combining the upper and lower derivatives, one can describe a situation where the polymer deformation partially follows the flow, making it non-affine to a certain degree. This type of constitutive relation is of interest, e.g. to capture the formation of shear bands as observed in worm-like micellar solutions [66–68]. It will turn out that this class of model does not converge to any elastic solid, even when taking the limit *λ* → ∞.

To show this, we consider the derivative used, for example, in the Johnson–Segalman model [69], which takes into account the possibility that the polymer does not follow the flow of the solvent in an affine fashion but slips with respect to the flow. This is accomplished by introducing the polymer velocity **v**_*a*_, which satisfies

3.6 $$\nabla {\text{v}}_{a}=\frac{a}{2}[\nabla \text{v}+{\left(\nabla \text{v}\right)}^{\text{T}}]+\frac{1}{2}[\nabla \text{v}-{\left(\nabla \text{v}\right)}^{\text{T}}]=\frac{1+a}{2}\nabla \text{v}-\frac{1-a}{2}{\left(\nabla \text{v}\right)}^{\text{T}},$$

where *a* (the so-called slip parameter) satisfies −1 ≤ *a* ≤ 1. Indeed, for *a* = 1, $\nabla {\text{v}}_{a}$ and ∇v are the same, and the polymer follows perfectly. This is no longer the case for *a* ≠ 1. The antisymmetric parts of $\nabla {\text{v}}_{a}$ and ∇v are the same, which means that **v**_*a*_ and **v** have the same vorticity, so that the polymer follows any solid body rotation of the flow perfectly. On the other hand, the rate of deformation of the polymer (symmetric part) satisfies ${\dot{\gamma }}_{a}=a\dot{\gamma }$. One can now define the upper convected derivative with respect to the slipping polymer,

3.7 $${\overset{▽}{\left(\text{A}\right)}}_{a}\equiv \frac{\text{d}\text{A}}{\text{d}t}-{\left(\nabla {\text{v}}_{a}\right)}^{\text{T}}\cdot \text{A}-\text{A}\cdot (\nabla {\text{v}}_{a})=\frac{1+a}{2}\overset{▽}{\text{A}}+\frac{1-a}{2}\overset{△}{\text{A}},$$

where in the following d/d*t* denotes the material derivative. This resulting superposition of upper and lower derivatives gives rise to the so-called Gordon–Schowalter derivative [70].

To illustrate the consequences of this non-affine motion, we consider a relaxation law based on (3.7), as is used, for example, in the Johnson–Segalman model. In that case, the evolution equation in the limit *λ* → ∞ takes the form

3.8 $${\overset{▽}{\left(\text{A}\right)}}_{a}=0.$$

Importantly, unless *a* = ±1, this equation in general does not have an explicit integral in terms of **F**, as in (3.2). We will see that this points to the absence of an elastic correspondence. To illustrate this, we consider the specific case of a uniform, steady shear flow $\text{v}=\dot{\gamma }y{\text{e}}_{x}$, and take the initial conditions as **A** = **I**. Solving (3.8) for this velocity field, one obtains [3,71]

3.9 $$\text{A}=\left(\begin{array}{cc} \frac{1}{\left(1-a\right)}\left[1-a\cos\left(\sqrt{1-a^{2}}\dot{\gamma }t\right)\right] & \frac{a}{\sqrt{1-a^{2}}}\sin\left(\sqrt{1-a^{2}}\dot{\gamma }t\right)\\ \frac{a}{\sqrt{1-a^{2}}}\sin\left(\sqrt{1-a^{2}}\dot{\gamma }t\right) & \frac{1}{\left(1+a\right)}\left[1+a\cos\left(\sqrt{1-a^{2}}\dot{\gamma }t\right)\right] \end{array}\right).$$

Hence, **A** exhibits an oscillatory behaviour when *a*^2^ ≠ 1. Such an oscillatory response during a simple shear deformation cannot correspond to any elastic model as defined by (2.15).

Physically, the oscillations can be understood from the non-affine kinematics described by (3.6). The flow $\text{v}=\dot{\gamma }y{\text{e}}_{x}$ can be written as a superposition of an elongational flow and a rigid body rotation of equal amplitude. Any slip (*a* < 1) removes part of the elongation flow, while the full rigid body rotation is retained. This effectively leads to an ‘excess’ rigid body motion, which gives rise to a periodic ‘flow’ of the polymer with a frequency $\sqrt{1-a^{2}}\dot{\gamma }$. We remark that these oscillations have a purely kinematic origin, and thus persist for a Johnson–Segalman fluid that is sheared at finite values of *λ* [4].

From these observations we draw an important conclusion. Only in the cases where *a*^2^ = 1 do the viscoelastic constitutive relations exhibit a well-defined elastic limit, in the sense that their behaviours converge to that of an elastic solid in the limit of *λ* → ∞. The very same conclusions were reached in the context of emulsions, whose drops deform into ellipsoids—in that case the eigenvalues of **A** represent the square of the semi-axes of the deformed droplets [59,60]. Any other time derivative, which implies non-affine motion, does not correspond to any limit of the theory of elasticity. As an example, we have seen that simple shear leads to oscillations in **A**, which cannot represent any rubber-like behaviour, even in the absence of any relaxation process.

### (c) Large Deborah number versus large Weissenberg number

Up to now we have considered the limit *λ* → ∞, without specifying under what conditions the time scale *λ* can be considered sufficiently large. A distinction should be made between a high-frequency response at small amplitude of deformation and a low-frequency response at large deformation [2,3,72]. The former is governed by the Deborah number, De = *λω*, where *ω* is a typical frequency at which the material is excited during unsteady dynamics. The latter is governed by the Weissenberg number, $\mathrm{Wi}=\lambda \dot{\gamma }$, where $\dot{\gamma }$ is a typical imposed shear rate (which can be constant in time).

#### (i) Shear flow in the Johnson–Segalman model

We now make explicit the different roles of large De and large Wi, and what it means to consider *λ* → ∞. For this, we again consider the simple shear problem, but now at finite *λ*. As a model, we take the Johnson–Segalman fluid, defined as

3.10 $$\sigma_{p}=a\mu (\text{A}-\text{I}),\;{\overset{▽}{\left(\text{A}\right)}}_{a}=-\frac{1}{\lambda }(\text{A}-\text{I}).$$

The polymer stress has a neo-Hookean structure, with the relaxation based on the Gordon–Schowalter derivative. Combining the two equations, the model can be recast in the form of a Maxwell fluid based on a non-affine derivative,

3.11 $$\sigma_{p}+\lambda {\left({\overset{▽}{\sigma }}_{p}\right)}_{a}=\eta_{p}\dot{\gamma },$$

where *η*_*p*_ = *a*^2^*μλ*. We remark that the effect of *a* only couples to the nonlinear term of the time derivative (this follows from inspecting the definition). Hence, the Johnson–Segalman model at small deformations is independent of the slip paramater *a*: this is commonly referred to as linear viscoelasticity. Non-affine effects only appear at large deformations.

Considering a simple shear flow with a stress-free initial condition (and omitting fluid inertia), the system (3.10) can be solved and gives for the shear component [66]

3.12 $$A_{xy}=\frac{a\;\text{Wi}}{1+(1-a^{2}){\text{Wi}}^{2}}\left(1-{\text{e}}^{-(t/\lambda )}\left[\cos(\sqrt{1-a^{2}}\;\dot{\gamma }t)-\text{Wi}\sqrt{1-a^{2}}\sin(\sqrt{1-a^{2}}\;\dot{\gamma }t)\right]\right).$$

There are now two distinct ways of taking the limit *λ* → ∞. In the first, we consider *t* ≪ *λ*, such that the polymer did not yet have any time to relax. Formally, this corresponds to Wi → ∞ at finite *t*. In this case (3.12) indeed converges to *A*_*xy*_ as given by (3.9). In the second limit, we first consider large times *t* ≫ *λ* at finite Wi, and subsequently send Wi → ∞. In this case, one can omit the exponential term in (3.12) and recover a steady-state response that depends on Wi. This steady-state response of the polymer is non-monotonic with Wi and has therefore been used to describe shear banding [66–68]. Transiently, when *t* ∼ *λ*, one observes damped oscillations that give rise to an overshoot of stress, as is well known for the Johnson–Segalman model [71]. As far as we are aware, however, these oscillations have not previously been recognized as a signature that such a viscoelastic model cannot possess an elastic limit.

#### (ii) The Pipkin diagram: affine versus non-affine motion

**Figure 4.**
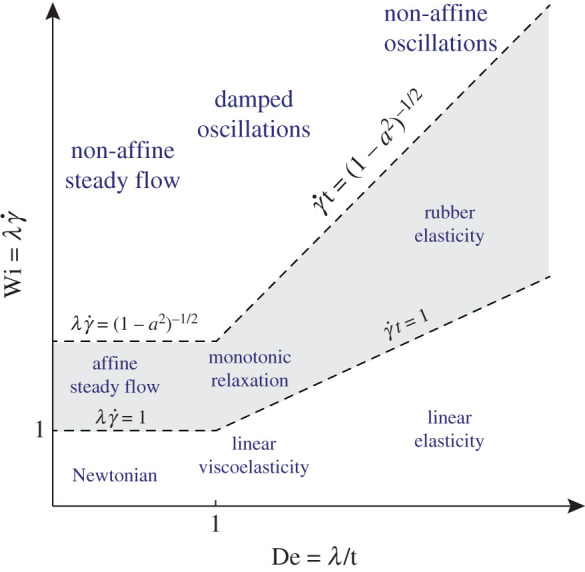
Pipkin diagram for the polymer response under simple shear. The axes report $\mathrm{Wi}=\lambda \dot{\gamma }$ and De = *λ*/*t*, where *t* is the time after starting the flow from a stress-free initial condition. The appearance of damped oscillations for *a*^2^ ≠ 1 is associated with the absence of an elastic limit. The figure summarizes the regimes of the Johnson–Segalman model (3.12), but the result is valid for arbitrary polymer models under shear.

In the present case of steady shear flow, we do not impose any oscillatory motion, so in a sense it could be considered as a case of vanishing Deborah number. However, given that at *t* = 0 we start from a stress-free initial condition, *t*^−1^ effectively provides a frequency of excitation of the polymer: we therefore use De = *λ*/*t* to characterize the unsteady response. Then, the two distinct limits discussed above correspond, respectively, to the limit Wi → ∞ at finite De and to the limit De → ∞ at finite Wi. We proceed by summarizing the response during simple shear in the form of a ‘Pipkin diagram’, in figure 4, resembling those found in [3,5].

The horizontal and vertical axes in figure 4, respectively, indicate the separate roles of De = *λ*/*t* and $\mathrm{Wi}=\lambda \dot{\gamma }$. Since $\dot{\gamma }t$ is a typical strain, the line Wi = De delineates small from large deformations. Below the line, one finds linear elasticity (high De) and linear viscoelasticity (intermediate De), both pertaining to small deformations. At low De, one reaches the limit of steady flow, where at long times the deformations will become large. The corresponding behaviour at small Wi is obviously Newtonian, while nonlinear viscoelastic effects (such as normal stress differences) appear at intermediate Wi.

In the traditional Pipkin diagram, no distinction is made between the affine and non-affine responses. Here we emphasize that the behaviour in the elastic limit Wi, De → ∞, crucially depends on the (non-)affine nature of the polymer motion. In particular, (3.12) shows that oscillations emerge at a frequency $\dot{\gamma }\sqrt{1-a^{2}}$, which are damped over a time scale *λ*. This emergence of (transient) oscillatory motion is the hallmark of non-affine effects—and signals the absence of an elastic limit. Only when *a*^2^ = 1, corresponding to perfectly affine motion, does Wi, De → ∞ give the response of an elastic solid.

### (d) Dynamics of viscoelastic solids

It is important to emphasize that figure 4 only pertains to the polymer stress **σ**_*p*_. In the presence of a viscous solvent (as in the Oldroyd-B model) the high-frequency stress response is actually dictated by the solvent viscosity, not by the polymer elasticity. Still, the limit *λ* = ∞ of the Oldroyd-B fluid is very useful for describing *viscoelastic solids*. By this, we refer to materials that perfectly recover their reference state once all stress is released, but at the same time exhibit dissipation during transient deformations. A schematic of a viscoelastic solid is obtained from figure 3 by omitting the dashpot in the upper branch, in which case one recovers the Kelvin–Voigt solid [73]. In the tensorial constitutive equation this corresponds precisely to taking the limit *λ* = *η*_*p*_/*μ* → ∞, while keeping a finite ratio *η*_*s*_/*μ*. In this limit, the Oldroyd-B fluid thus reduces to a neo-Hookean solid in parallel with a Newtonian solvent.

The effect of dissipation is indeed extremely relevant for soft rubbers and elastomers. As a prime example we mention pressure-sensitive adhesives, whose adhesive strength is enhanced through strong dissipation during debonding [29–31]. It should be noted that such adhesives undergo irreversible deformations and do not recover their original reference state—as such they are not described by viscoelastic models in the limit *λ* → ∞. However, there is a growing interest in the so-called reversible adhesives [74–77]. These do remain intact after debonding and as such fall into the class of viscoelastic solids that preserve their reference state. Another important example involving viscoelastic solids is found when liquid drops spread over soft elastomeric substrates [27,28,78], which was shown in figure 1*b*. The spreading dynamics of such drops is known to be extremely slow because of dissipation inside the solid. This phenomenon is called *viscoelastic braking* [25,26], and much research is currently dedicated to finding appropriate models for this coupled fluid–structure interaction problem [28,79,80].

The present analysis demonstrates that (reference state-preserving) viscoelastic solids can indeed be captured by a fully Eulerian approach, through viscoelastic fluids (like Oldroyd-B) in the limit *λ* → ∞. By now, it should be clear that this route only works for affine models, with an upper convected derivative for the conformation tensor.

### (e) A brief note on constitutive models without the conformation tensor

The discussion so far has been restricted to constitutive relations based on the conformation tensor **A**, the reason being the elegant connection between **A** and the Finger tensor **B** in the theory of elasticity. A natural question is whether the above considerations carry over to other viscoelastic theories, which do not explicitly involve the conformation tensor. Specifically, many models are based on the idea of expressing stress directly as a functional of the history of deformation [5,9]. Here we briefly touch upon such models, by expanding on the discussion of the upper convected Maxwell model.

#### (i) Integral forms

We first discuss the integral formulation of viscoelasticity, which is based on a memory kernel acting on past deformations. The approach is illustrated through a direct integration of the upper convected Maxwell model, as given by (2.7). The integration was already achieved for *λ* = ∞ (see the discussion around (3.1)), but the integral exists also at finite *λ* [3]. For a stress-free initial condition at *t* = 0, the solution reads^3^

3.13 $$\text{A}(t)={\text{e}}^{-(t/\lambda )}\text{B}(t)+\frac{1}{\lambda }\int_{0}^{t}\;\text{d}t^{\prime }\;{\text{e}}^{-(t-t^{\prime })/\lambda }{\text{B}}^{t^{\prime }}(t),$$

where we introduced

3.14 $${\text{B}}^{t^{\prime }}(t)=\text{F}(t)\cdot [{\text{F}}^{-1}(t^{\prime })\cdot {\text{F}}^{-T}(t^{\prime })]\cdot {\text{F}}^{\text{T}}(t).$$

The interested reader can find the derivation in appendix B. In this review, we focus on materials with a single time scale only. This can easily be generalized to multiple modes [2], with separate time constants *λ*_*i*_. In the limit of a continuous, broad distribution of time scales, one arrives at power-law materials [13,51,52] for which the exponential kernel of (3.13) is replaced by a power law.

The object **B**^*t*′^(*t*) can be seen as a generalization of the Finger tensor: while **B**(*t*) ≡ **B**^0^(*t*) measures the stretches compared with a ‘reference’ state at *t* = 0, the tensor **B**^*t*′^(*t*) measures the stretches at time *t* compared with the state at another time *t*′. The elastic correspondence is easily recovered in the integral formalism. Taking *λ* → ∞ at finite *t*, (3.13) reduces to **A**(*t*) = **B**(*t*) at all times *t* ≥ 0. For viscoelastic fluids with a finite *λ*, the initial condition plays no specific role and it is more natural to express (3.13) as

3.15 $$\text{A}(t)=\frac{1}{\lambda }\int_{-\infty }^{t}\;\text{d}t^{\prime }\;{\text{e}}^{-(t-t^{\prime })/\lambda }{\text{B}}^{t^{\prime }}(t).$$

Once multiplied by the shear modulus, to obtain **σ**_*p*_, this form goes by the name of the Lodge equation [3]. This form nicely reveals that the stress can indeed be considered as an integral over the entire history of deformation. The associated kernel exp ( − (*t* − *t*′)/*λ*) accounts for the fading memory over a time *λ*.

A more general integral formulation of viscoelasticity goes back to the Kaye–Bernstein–Kearsly–Zapas model (KBKZ), which is directly inspired by the theory of elasticity [53,55]. In this model, the non-Newtonian contribution to the stress is written as

3.16 $$\sigma_{p}=\int_{-\infty }^{t}\;\text{d}t^{\prime }\;[W_{1}(t-t^{\prime }){\text{B}}^{t^{\prime }}(t)-W_{2}(t-t^{\prime }){\left({\text{B}}^{t^{\prime }}(t)\right)}^{-1}].$$

Here the connection to the elasticity theory is very explicit: the stress is of the same form as (2.15), with time-dependent elastic moduli *W*_1_, *W*_2_ that serve as memory kernels. The Lodge equation (3.15) is recovered by *λW*_1_ = *μ*exp ( − (*t* − *t*′)/*λ*) and *W*_2_ = 0. Let us remark that, in the general case where both kernels *W*_1_(*t* − *t*′) and *W*_2_(*t* − *t*′) are non-zero, the KBKZ model cannot be reduced to a simple conformation tensor description. We can interpret the integral over **B**^*t*′^(*t*) as the conformation tensor **A** using (3.15). However, the integral over [**B**^*t*′^(*t*)]^−1^ does not lead to **A**^−1^, not even when both kernels have the exact same time dependence. Hence, the deformation history in (3.16) gives rise to two independent ‘state variables’ (or two conformation tensors) that determine the polymer stress.

#### (ii) Expansions

The upper convected Maxwell model can thus be expressed in various equivalent forms: the conformation tensor description, (2.4) and (2.7), the integral (3.16), but also of course in the more common differential from (2.8). This, however, is not the case for all viscoelastic models, in particular when the models result from expansions. For example, the Oldroyd-B model can be seen as a special case of Oldroyd’s eight-constant model [2,50],

3.17 $$\sigma +\lambda_{1}\overset{▽}{\sigma }+\lambda_{2}(\dot{\gamma }\cdot \sigma +\sigma \cdot \dot{\gamma })+\lambda_{3}\text{tr}(\sigma )\dot{\gamma }+\lambda_{4}(\sigma :\dot{\gamma })\text{I}=\eta [\dot{\gamma }+\lambda_{5}\overset{▽}{\dot{\gamma }}+\lambda_{6}\dot{\gamma }\cdot \dot{\gamma }+\lambda_{7}(\dot{\gamma }:\dot{\gamma })\text{I}].$$

Equation (3.17) is an expansion that contains all quadratic terms in stress and strain rate, provided they satisfy frame invariance. The Oldroyd-B and Johnson–Segalman fluids are particular versions of it, with *η*_*p*_, *η*_*s*_, *μ* and *a* as the only non-zero constants. As far as we are aware, however, the general eight-constant model cannot be reduced to a description in terms of a conformation tensor, given by some expression **σ**_*p*_(**A**) and a relaxation equation for **A**. In its most general form, the eight-constant model therefore does not converge to an elastic solid when *λ* → ∞.

Another special case of the Oldroyd eight-constant model is the so-called second-order fluid [5,50], defined by the constitutive equation

3.18 $$\sigma_{p}=b_{2}\overset{▽}{\dot{\gamma }}+b_{11}\dot{\gamma }\cdot \dot{\gamma }.$$

This results from the so-called Rivlin–Ericksen expansion, which gradually builds in memory of past deformations. The quadratic terms in (3.18) are the lowest order to give non-Newtonian effects. Indeed, the second-order fluid cannot be represented in terms of a conformation tensor. This can be seen by considering the case where the flow is suddenly stopped at some time *t*_0_, so that $\dot{\gamma }=0$ for *t* > *t*_0_. Evaluating the polymer stress in (3.18), we find that **σ**_*p*_ = **0** for *t* > *t*_0_. Hence any stress present at *t*_0_ is instantaneously relaxed, which is incompatible with a gradually relaxing conformation tensor. By the same argument it is also clear that the second-order fluid has no limit in which it can converge to solid-like behaviour.

Let us emphasize that the absence of an elastic correspondence should not be seen as a shortcoming of a model. In the case of the second-order fluid, the perturbative expansion was not designed to capture strongly unsteady effects, but rather to capture nearly steady flows. For example, in spite of its simplicity, (3.18) exhibits normal stress differences in shear flow, and successfully captures various viscoelastic phenomena [81–83]. Similarly, the Johnson–Segalman model was never intended to describe elastic solids, but rather to capture non-monotonic stress relaxation.

## 4. Collapse of a cylinder under surface tension

We now illustrate the importance of the elastic correspondence through the collapse of a (visco)elastic cylinder under surface tension. As was discussed in the Introduction, figure 1*d* shows the capillary instability for an elastic solid that consists of a cross-linked agar gel [20]. It is cross-linked to such a degree that the gel possesses (and maintains) a reference state, which ultimately prevents the break-up of the thin threads. The structures that appear, in particular the thin threads, strongly resemble those seen during the capillary break-up of viscoelastic liquids that do not possess a reference state [21,45,84]. Figure 1*e* shows the break-up of a water jet containing a low concentration of a high molecular flexible polymer, with a relaxation time of about 0.01 s. The break-up process repeats itself periodically in space but with a time delay in between, so one can see an almost cylindrical thread at different stages of thinning. An alternative geometry is that of a liquid bridge between two plates, which leads to a single thread. In each case, the thread radius is observed to thin exponentially in time [21,84,85]. As another amusing example of the interaction between elasticity and capillarity, albeit in a different context, we mention the interaction of an elastic beam with a liquid drop [86].

Below we will first re-derive the classical result of exponential thinning of viscoelastic fluids, and show how the elastic correspondence is actually a key element in solving the problem. Subsequently, the usefulness of the elastic correspondence is underlined by numerical solutions of viscoelastic *solids*, by means of an Oldroyd-B fluid in the limit *λ* → ∞. The result is compared with a purely elastic simulation of a neo-Hookean solid. Finally, it is shown that the collapse changes dramatically when the model does not exhibit an elastic correspondence, as is exemplified by the Johnson–Segalman fluid.

### (a) Viscoelastic fluid

The capillary thinning of an infinitely long liquid cylinder is due to an elongational flow, defined by the Eulerian velocity field (3.4). From a Lagrangian perspective, this can be seen as a stretching of the cylinder by a rate $\dot{\epsilon }$, and a lateral contraction dictated by volume conservation. Denoting the cylinder radius by *h*(*t*), the radially inward flow implies $\dot{h}=-(1/2)h$, so that

4.1 $$h=h_{0}\;{\text{e}}^{-(1/2)\dot{\epsilon }t}.$$

The goal is to determine the value of $\dot{\epsilon }$ and the constant *h*_0_ (which is not equal to the initial thread radius *R*_0_) for the process of capillary thinning.

#### (i) Stretch and relax

We consider the conformation tensor **A** in the Oldroyd-B fluid during the thinning, determined by (2.7) with elongational flow. For a rubber band (*λ* → ∞) the conformation tensor would simply follow the Finger tensor, which is given by (3.5). The general solution with finite relaxation time was already given in integral form in (3.13). One verifies that $B_{zz}^{t^{\prime }}(t)=\exp(2\dot{\epsilon }(t-t^{\prime }))$, representing the exponential separation over a time lag *t* − *t*′. With this, the integral (3.13) gives the axial component of the conformation tensor

4.2 $$A_{zz}(t)=\frac{2\dot{\epsilon }\lambda }{2\dot{\epsilon }\lambda -1}\;{\text{e}}^{(2\dot{\epsilon }-(1/\lambda ))t}-\frac{1}{2\dot{\epsilon }\lambda -1},$$

and similarly one finds for the radial component

4.3 $$A_{rr}(t)=\frac{\dot{\epsilon }\lambda }{\dot{\epsilon }\lambda +1}\;{\text{e}}^{-(\dot{\epsilon }+(1/\lambda ))t}+\frac{1}{\dot{\epsilon }\lambda +1}.$$

The solution (4.2) very nicely brings out the competition between stretching and relaxation. The term $\exp(2\dot{\epsilon }t)$ reflects the stretching of the polymer by the flow; at the same time, the polymer relaxation reduces the stretch as exp ( − *t*/*λ*). When $\dot{\epsilon }\lambda >1/2$, as will be the case in capillary thinning, the exponential stretching dominates over the relaxation. When $\dot{\epsilon }\lambda <1/2$, the relaxation is strong enough that *A*_*zz*_ saturates at some finite value.

The value of $\dot{\epsilon }$ can be found when demanding that the polymer stress **σ**_*p*_ ∼ *μA*_*zz*_ has the same time dependence as the capillary pressure $\gamma /h(t)∼\exp(\dot{\epsilon }t/2)$. Equating this exponential to that in (4.2), one finds $\dot{\epsilon }\lambda =2/3$. One thus concludes that the thinning of the thread scales as *h* ∼ exp ( − *t*/3*λ*), a result that goes back to Entov [87].

#### (ii) The elastic correspondence

Figure 2 shows numerical simulations of the break-up in the Oldroyd-B fluid [48]. For finite *λ*, we indeed observe the expected exponential thinning dynamics (blue line). However, the initial stage of the break-up (on the scale of the capillary time *τ*) is not exponential; in fact it closely follows the Newtonian break-up, *λ* = 0, plotted as the purple line. In this early regime, the polymer is not yet sufficiently stretched to compete with capillary forces, and we observe a near independence of *λ*. In the elastic limit *λ* = ∞ (red line), the thread does not go to zero at all, but saturates at a finite thickness. This corresponds to a neo-Hookean solid, and, for example, describes the cross-linked agar gel of figure 2*a*.

So how can we compute the prefactor *h*_0_ of the thinning law (4.1)? For this, we make use of the elastic correspondence. Upon inspection of figure 2, one can infer that *h*_0_ is essentially given by the final thickness of the purely elastic thread (this becomes exact when *τ* ≪ *λ*). This final thickness follows from an elasto-capillary stress balance. The elastic stress is simply that of a neo-Hookean rubber band, with elongation stretch given by (*R*_0_/*h*)^2^, where *R*_0_ is the initial cylinder radius. When sufficiently soft, the elastic stress scales as ∼*μ*(*R*_0_/*h*)^4^. Balancing this with the capillary stress *γ*/*h*, one obtains the elasto-capillary length [45]

4.4 $$\ell_{e}={\left(\frac{\mu R_{0}^{4}}{\gamma }\right)}^{1/3}.$$

A detailed analysis matching the cylinder to a large drop shows that the exact prefactor in (4.1) reads *h*_0_ = ℓ_*e*_/2^1/3^ [48].

The elastic correspondence goes much beyond computing *h*_0_. Using a lubrication description, it was conjectured by Entov & Yarin [44] and confirmed in [21,84] that the entire *shape* of the thinning thread—at finite *λ*—could be described by that of the corresponding elastic solid. This is a scheme that we recently confirmed to be true in general, beyond the lubrication description [48].

### (b) Viscoelastic solid

We argued in §3d that the elastic correspondence allows us to model viscoelastic solids as an Oldroyd-B fluid, taking the limit *λ* → ∞. This idea is tested by two distinct numerical schemes to compute the capillary collapse. We first consider an Eulerian simulation for the Oldroyd-B fluid with infinite relaxation time, followed by a Lagrangian simulation of a neo-Hookean elastic cylinder. The final state should be the same because of the elastic correspondence, but there is an important difference: because of the solvent viscosity, the Oldroyd-B model is able to capture the dynamics of the solid in the presence of viscous damping.

#### (i) The Oldroyd-B fluid as a viscoelastic solid

We simulate the Oldroyd-B equations (2.1), (2.2), (2.8) in the limit of *λ* → ∞, taken such that *μ* = *η*_*p*_/*λ* remains finite. Then the polymeric stress is governed by ${\overset{▽}{\sigma }}_{p}=0$. The collapse is driven by surface tension, and the stress boundary condition at the free surface is

4.5 n⋅σ=−γκn,

where

4.6 $$\kappa =\frac{1}{h{\left(1+h_{z}^{2}\right)}^{1/2}}-\frac{h_{zz}}{{\left(1+h_{z}^{2}\right)}^{3/2}},\;\text{n}=\frac{{\text{e}}_{r}-{\text{e}}_{z}h_{z}}{{\left(1+h_{z}^{2}\right)}^{1/2}}$$

are (twice) the mean curvature and the surface normal, respectively. If *h*(*z*, *t*) is the thread profile, the kinematic boundary condition becomes

4.7 $$\frac{\partial h}{\partial t}+u_{z}(z,h)\frac{\partial h}{\partial z}=u_{r}(z,h),$$

where **v** = *u*_*r*_**e**_*r*_ + *u*_*z*_**e**_*z*_ in cylindrical coordinates. As an initial condition, we take the free surface shape

4.8 $$h(z,0)\equiv h_{0}(z)=R_{0}\left[1-\epsilon \cos\left(\frac{z}{2R_{0}}\right)\right],$$

and the velocity field vanishes initially; boundary conditions are periodic. First, we will consider the case that stresses vanish initially. To illustrate the predictions of (3.3), we will then consider an initial uniform axial stress.

We have carried out a simulation for a fluid cylinder of radius *R*_0_, which is slightly perturbed according to (4.8) with *ϵ* = 0.05. Material parameters are fixed by dimensionless numbers $\eta_{s}/\sqrt{\rho \gamma R_{0}^{3}}=0.79$ and *μR*_0_/*γ* = 0.0119. In order to calculate the interface evolution accurately, we apply the boundary fitted coordinate method, where the liquid domain is mapped onto a rectangular domain through a coordinate transformation. The hydrodynamic equations are discretized in this domain using fourth-order finite differences, with 22 equally spaced points in the radial direction and 1000 equally spaced points in the axial direction. An implicit time advancement is performed using the second-order backward finite differences with a fixed time step $0.05\sqrt{\rho R_{0}^{3}/\gamma }$; details of the numerical procedure can be found elsewhere [88].

**Figure 5.**
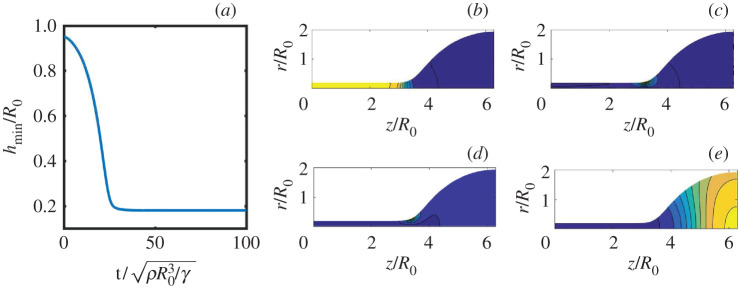
(*a*) Time evolution of the minimum thread radius for the viscoelastic solid, simulated by an Oldroyd-B fluid with *λ* = ∞. Parameters are *ϵ* = 0.05, $\eta_{s}/\sqrt{\rho \gamma R_{0}^{3}}=0.79$ and *μR*_0_/*γ* = 0.0119. (*b*–*e*) Polymeric stresses in the final state of the fluid simulation: (*b*) *σ*_*p*,*zz*_; (*c*) *σ*_*p*,*zr*_; (*d*) *σ*_*p*,*rr*_; (*e*) *σ*_*p*,*θθ*_. The purple to yellow (dark to light) colour gradient represents the change from minimum to maximum stress, respectively. (Online version in colour.)

We begin with the case where there is no stress in the initial condition. In figure 5*a*, we show the minimum thread radius *h*_min_ as a function of time. As the bridge collapses, elastic stress builds up until it is balanced by surface tension and *h*_min_ approaches a constant value, as shown in figure 5. At this point, the solution becomes stationary, time derivatives vanish and the velocity goes to zero. As a result, the solvent viscosity does not affect the final state, which should be identical to that of a neo-Hookean solid. However, the Oldroyd-B simulation also captures the transient dynamics of the viscoelastic solid. For completeness, figure 5*b*–*d* shows the different components of the stress tensor in the static final state. The axial stress *σ*_*p*,*zz*_ is highest inside the thread, where fluid elements are stretched the most in the axial direction. Radial stresses *σ*_*p*,*rr*_, on the other hand, are most pronounced inside the drop, where fluid elements are stretched in the radial direction.

#### (ii) The neo-Hookean solid

We now calculate the steady state of an elastic neo-Hookean material using nonlinear elasticity, as described by

4.9 σ=μ(B−I)−pI,

and subject to the incompressibility constraint J=det(F)=1. First we discuss the case without pre-stress. The pressure *p* is adjusted such that *J* = 1 is satisfied. Instead of a dynamical equation, the condition for static equilibrium reads ∇⋅σ=0, i.e. (2.1) with **v** = **0**, with the elasto-capillary boundary condition (4.5).

**Figure 6.**
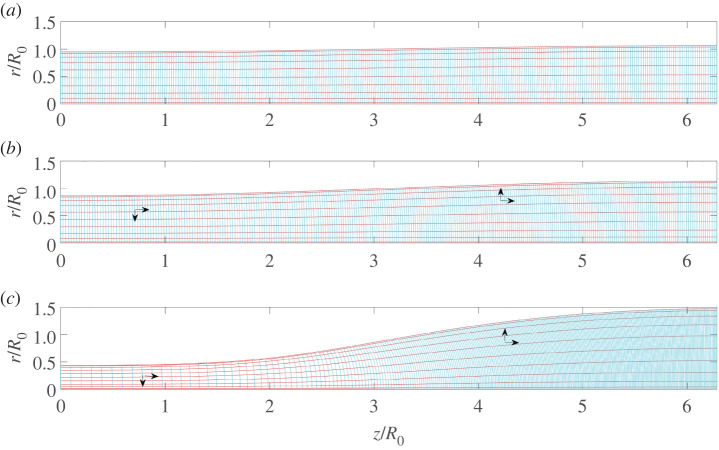
The elastic simulation: (*a*) the reference state, *μR*_0_/*γ* = ∞; (*b*) *μR*_0_/*γ* = 0.2; (*c*) *μR*_0_/*γ* = 0.1. Red (cyan) lines describe constant values of *η* (*ξ*). The arrows indicate the direction of the mesh deformation. (Online version in colour.)

To determine the final state of the collapsed cylinder, we solve a nonlinear set of equations corresponding to the above conditions, based on a (stationary) mapping **x** = **x**(**X**), as illustrated in figure 6. To this end, we write the mapping in cylindrical coordinates: *r* = *r*(*R*, *Z*), *z* = *z*(*R*, *Z*). The coordinates *R* and *Z* are the radial and axial coordinates of the cylinder in the reference state. Using general formulae for **F** in cylindrical coordinates [89], incompressibility amounts to

4.10 $$\det\text{F}=\frac{r}{R}\left(\frac{\partial r}{\partial R}\frac{\partial z}{\partial Z}-\frac{\partial r}{\partial Z}\frac{\partial z}{\partial R}\right)=1,$$

while the stress can be computed from the Finger tensor

4.11 $$\text{B}=\text{F}\cdot {\text{F}}^{\text{T}}=\left(\begin{array}{ccc} \left({\left(\frac{\partial r}{\partial R}\right)}^{2}+{\left(\frac{\partial r}{\partial Z}\right)}^{2}\right) & 0 & \left(\frac{\partial r}{\partial R}\frac{\partial z}{\partial R}+\frac{\partial r}{\partial Z}\frac{\partial z}{\partial Z}\right)\\ 0 & {\left(\frac{r}{R}\right)}^{2} & 0\\ \left(\frac{\partial r}{\partial R}\frac{\partial z}{\partial R}+\frac{\partial r}{\partial Z}\frac{\partial z}{\partial Z}\right) & 0 & \left({\left(\frac{\partial z}{\partial Z}\right)}^{2}+{\left(\frac{\partial z}{\partial R}\right)}^{2}\right) \end{array}\right).$$

The solution depends on the dimensionless number *R*_0_*μ*/*γ*. In the ‘soft’ limit where the thread becomes very thin, *r* ≪ *R*_0_, the thickness of the thread scales as the elasto-capillary length scale ℓ_*e*_ given by (4.4).

**Figure 7.**
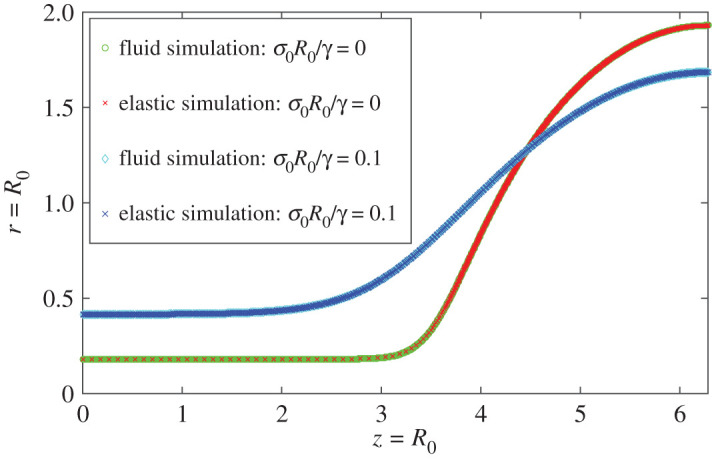
A comparison between the final shape of an elastic bridge driven by surface tension, using an Oldroyd-B fluid with *λ* = ∞, and a neo-Hookean elastic material. The elastic shear modulus is *μ* = *η*_*p*_/*λ* = 0.0119*γ*/*R*_0_. The green and red symbols (large deformation) represent vanishing initial stress, while to obtain the cyan and blue symbols (small deformation) the bridge contained a uniform initial axial stress *σ*_0_. In each case, the results are virtually identical. (Online version in colour.)

To solve the problem numerically, we define the reference state by

$$R=h_{0}(\xi )\eta \;\text{and}\;Z=\xi ,$$

with the elastic domain defined by *η* ∈ [0, 1] and *ξ* ∈ [0, 2*πR*_0_]; *h*_0_ is once again defined by (4.8) and *ϵ* = 0.05. We are looking for two unknown functions *f* and *g*, where *r* = *r*(*R*, *Z*) = *f*(*η*, *ξ*) and *z* = *z*(*R*, *Z*) = *g*(*η*, *ξ*), as well as the pressure *p*(*η*, *ξ*). These three unknowns are found from solving the three equations (4.10), (2.1) at steady state and with boundary conditions (4.5). The free surface *h*(*z*) then is given by the parametric representation *h*(*g*(1, *ξ*)) = *f*_1_(1, *ξ*), from which the curvature *κ* can be evaluated. In figure 6, the domain is discretized using fourth-order finite differences with 301 equally spaced points in the *ξ* direction and 11 Chebyshev collocation points in the *η* direction. For the results presented in figure 7, a finer mesh was used with 2001 equally spaced points in the *ξ* direction. The resulting system of nonlinear equations is solved using a Newton–Raphson technique [88]. We solve the problem by starting with the reference state as the initial guess and *μR*_0_/*γ* sufficiently large (*μR*_0_/*γ* = 100) to ensure the convergence of the Newton–Raphson iterations. Once we get a solution, we use this solution in a new run with a smaller value of *μR*_0_/*γ*.

The result is shown in figure 6, which describes the deformation of the mesh as well as of the free surface, for various values of *μR*_0_/*γ*. The resulting shapes closely match those in figure 2 at moderate stiffness, and agree with simulations in [46]. In figure 7, we compare the elastic equilibrium state (red crosses) with the stationary state reached in the simulation of the Oldroyd-B model for *μR*_0_/*γ* = 0.0119 (green circles). The agreement is perfect—illustrating that, for this problem where a stationary state is approached, the Oldroyd-B fluid in the limit of *λ* → ∞ converges to a neo-Hookean solid. A detailed similarity analysis of this problem is provided in [48].

To further test (3.3) in the case of a pre-stressed material, we repeat the same analysis for both the fluid and the elastic case, but assuming an initial purely axial stress. This means that the fluid equations are solved with the initial condition *σ*_*p*,*zz*_ = *σ*_0_, and are solved until a new stationary state is reached. As for the elastic simulation, (3.3) is used with $\sigma_{p,zz}^{\left(0\right)}=\sigma_{0}$. As seen in figure 7 (cyan diamonds, fluid simulation; blue crosses, elastic simulation), the equilibrium is now reached at a much larger value of *h*_min_, because less of a build-up of elastic stresses is needed to counter surface tension. Once more, there is perfect agreement with the Oldroyd-B fluid simulation, illustrating the general formula (3.3) for arbitrary initial conditions.

Thus, we have demonstrated two very different numerical solutions to the same problem of finding the equilibrium shape of an elastic thread constricted by surface tension, one being Eulerian and the other Lagrangian. This represents a satisfying confirmation of the equivalence of fluid flow and nonlinear elasticity in the limit of infinite relaxation time, and presents a valuable check on the stability and reliability of the underlying numerical methods.

### (c) The absence of an elastic limit: the Johnson–Segalman fluid

The above comparison was an illustration of our result, which assigns a unique elastic limit to the Oldroyd-B model for large relaxation times. The scenario changes dramatically in the presence of non-affine motion, for which there is no elastic correspondence. This will be explored for a Johnson–Segalman fluid with infinite relaxation time, characterized by ${\overset{▽}{\left(\text{A}\right)}}_{a}=0$. The other equations remain the same. Previous analysis of the long-wavelength limit has shown [45,90] that there can be no static solution for *a* < 1/2. Owing to the presence of non-affine slip, the amount of polymer stretch is not enough for the elastic stress to balance the capillary stress in a thinning cylinder. This means that the motion will not be arrested by surface tension, and the thread breaks up in finite time [45,90]. By contrast, the slender analysis predicts that for *a* > 1/2 the thread will not break up.

**Figure 8.**
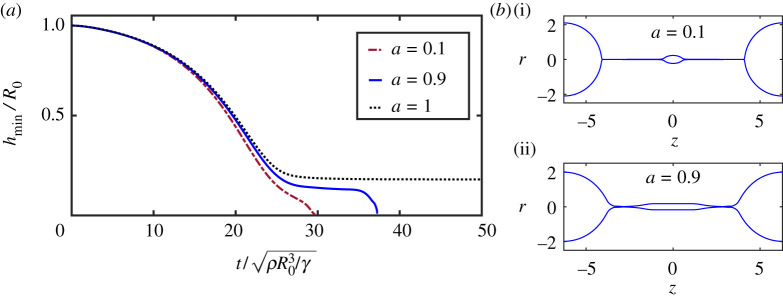
(*a*) The minimum thread radius *h*_min_ for a Johnson–Segalman fluid, as a function of time for different values of *a*. Only in the case of non-affine motion (*a* = 1) does the thread attain a static elastic solution. Break-up is observed for all values *a* ≠ 1. (*b*) Typical interface shapes for the collapse of a liquid bridge of Johnson–Segalman fluid close to break-up, for two values of the parameter *a*. The case *a* = 0.1 corresponds to *t* = 29.7747 and *h*_min_ = 0.000886, while for *a* = 0.9, *t* = 37.271 and *h*_min_ = 0.0124. (Online version in colour.)

Johnson–Segalman fluid simulations are carried out by integrating (3.10) with *λ* = ∞. We consider different values of *a* with the same numerical technique as described at the beginning of the section, using the same parameters *ϵ* = 0.05, $\eta_{s}/\sqrt{\rho \gamma R_{0}^{3}}=0.79$ and *μR*_0_/*γ* = 0.0119 as before. Figure 8*a* shows *h*_min_ as a function of time for three different values of the slip parameter *a*. As can be seen in the figure, the solution reaches a steady state only for the affine case *a* = 1 (black dotted line), as seen before in figure 5. In line with the lubrication analysis of [90], for *a* = 0.1 the thread thickness approaches zero linearly, and at a finite time (red dashed line). Remarkably, however, the case *a* = 0.9 also leads to break-up (blue solid line), a feature that was not predicted in the lubrication framework.

Figure 8*b* highlights further the behaviour of the pinching threads. Figure 8*b*(i) shows that for *a* = 0.1 the thread profile remains slender, and break-up is described by the similarity theory of Fontelos [90]. Figure 8*b*(ii) shows the case *a* = 0.9, which exhibits break-up despite the lubrication prediction that it should not. Indeed, the corresponding interface now develops a more complicated structure, with pinching near the drops, which violates the assumption of slenderness. We thus conclude that non-affine motion, for which the elastic limit does not exist, has a dramatic effect on the dynamics, in this case on the capillary collapse of a cylinder.

## 5. Energy and dissipation

There are striking dissimilarities in the approach to viscoelastic liquids and to elastic solids. We have seen in §2 that the constitutive relations in elasticity theory are defined by a free energy functional *W*, while the constitutive relations for viscoelastic liquids are most commonly expressed in terms of the stress tensor **σ**_*p*_. A fully conservative formulation of viscoelasticity is not possible, of course, since the process of relaxation involves dissipation. Still, a thermodynamic approach is very feasible, as described in Beris & Edwards [6]. This thermodynamic route was previously proposed by Leonov [54], and was formalized using the bracket [6] and the GENERIC formalisms [7]. We refer to Pasquali & Scriven [65] for a detailed discussion on how these theories are related.

The idea is again to describe the microstructure of complex fluids by a thermodynamic state variable, or order parameter, say **A**(**x**, *t*) in the case of the conformation tensor. The key element of the thermodynamic route is to associate an elastic free energy density with this field, *W*(**A**), which represents the free energy stored in stretched polymers. The formulation is then very similar to the theory of elasticity, which was based on *W*(**B**), except that one allows for dissipative processes via relaxation, expressed by $\overset{▽}{\text{A}}\neq 0$. An important advantage of the thermodynamic formulation of viscoelasticity is that, by construction, the constitutive relations are consistent with the laws of thermodynamics.

Here we further elaborate these ideas, and derive the explicit form of the energy equation for viscoelastic fluids. This serves two purposes. (i) It is surprisingly difficult to find general expressions for dissipation in common models of viscoelastic fluids [91]. In particular, we did not succeed in finding an energy equation for viscoelastic fluids that would generalize the usual energy conservation law for Newtonian flows. (ii) The thermodynamic formulation offers a very natural connection between elasticity and viscoelasticity, with the viscoelastic free energy *W*(**A**) playing the exact same role as the elastic free energy *W*(**B**). Rather than following the formalisms of statistical physics [6,7], we here offer a direct mechanistic route by separating the reversible and dissipative parts of the energy equation.

### (a) Flow versus relaxation

The order parameter formulation of viscoelasticity has the following ingredients:

(i) A symmetric rank-2 tensor-order parameter field **A**(**x**, *t*), which quantifies the stretched state of the polymer.
(ii) An elastic free energy density *W*(**A**), which is minimal for **A** = **I**.
(iii) A relaxation equation towards **A** = **I**, governing dissipation.

Since the conformation tensor now plays the role of a thermodynamic state variable, it is important to give it a proper definition. For microscopic bead–spring models, the conformation tensor can be expressed in terms of the (averaged) end-to-end vector of the dumbbell. To mimic the theory of elasticity, however, we here look for a purely continuum definition.

The quantification of the amount by which the polymer is stretched requires a comparison of the current state with a relaxed (isotropic) state at which the system is stress free. In a purely continuum description, the definition of **A** therefore requires a concept that can be characterized as an ‘instantaneous reference state’—this describes at each instant in time the state in which the polymer would be stress-free [5]. In a purely elastic material, devoid of relaxation, the reference state is the same at all times and can, for example, be chosen as the initial condition. For viscoelastic liquids, however, the memory of the initial condition is gradually fading. For instance, let us consider a case where, by applying appropriate constraint forces, we instantaneously stop the flow. The final state of the liquid will ultimately become stress-free, but this takes some time. In this situation, however, the current state of the liquid no longer evolves since the flow has stopped: it is the instantaneous reference state that will relax towards the liquid’s final state.

These ideas can be formalized upon introducing curvilinear material coordinates that describe the dynamics of material points following the flow. The curvilinear description allows us to properly define the conformation tensor and to distinguish its time evolution into a part due to ‘flow’ and a part that represents ‘relaxation’. We refer the reader to appendix A for details. Below we exploit this separation of flow and relaxation, which enables us to separate the storage of energy (stretching by flow) and its dissipation (via relaxation).

### (b) Stress, energy and dissipation

#### (i) Splitting the work: energy storage versus dissipation

With a continuum definition of **A** in place, we can find the correct structure of *W*(**A**) by borrowing the elastic energy *W*(**B**), as found on nonlinear elasticity. The energy must be a function of the invariants

5.1 $$I_{1}=A_{kk},\;I_{2}=\frac{1}{2}(A_{kk}^{2}-A_{ij}A_{ij})\;\text{and}\;I_{3}=\det(\text{A}),$$

with **A** taking the role of **B** in (2.12). There should be no confusion from using the same notation for the invariants of **A**. The choice of *W*(**A**) naturally determines the elastic limit, while the relaxation equation for **A** accounts for irreversible dissipation.

We now proceed to derive the expression for the stress and the dissipation, focusing first on *affine* polymer models. Given that we chose the conformation tensor **A** to express the stretching of the polymer, it must relax according to $\lambda \overset{▽}{\text{A}}=f(\text{A})$. The idea is that the reversible part of the deformation has the same form as the reversible change in free energy (2.11), so the remainder corresponds to dissipation. Writing the work in symmetric form $(1/2)\sigma_{p}:\dot{\gamma }$, and introducing the volumetric dissipation rate *ϵ*_*p*_, energy conservation requires that

5.2 $$\frac{1}{2}\sigma_{p}:\dot{\gamma }=\frac{\text{d}W}{\text{d}t}+\epsilon_{p},$$

where we have performed a split into reversible and irreversible parts: any work done during the deformation must be either stored in elastic energy or dissipated. With this convention, *ϵ*_*p*_ must be positive in order to be consistent with thermodynamics.

The time derivative d*W*/d*t* can be calculated using the definition of $\overset{▽}{\text{A}}$, yielding

5.3 $$\begin{array}{rl} \frac{\text{d}W}{\text{d}t} & =\frac{\partial W}{\partial \text{A}}:\frac{\text{d}\text{A}}{\text{d}t}=\frac{\partial W}{\partial \text{A}}:[{\left(\nabla \text{v}\right)}^{\text{T}}\cdot \text{A}+\text{A}\cdot (\nabla \text{v})+\overset{▽}{\text{A}}]\\ & =\left(\frac{\partial W}{\partial \text{A}}\cdot \text{A}\right):\dot{\gamma }+\frac{\partial W}{\partial \text{A}}:\overset{▽}{\text{A}}, \end{array}$$

where in the last line we made use of the symmetry of **A**. As anticipated in (5.2), this nicely separates into a term due to flow, proportional to $\dot{\gamma }$, and a term associated with the relaxation law, proportional to $\overset{▽}{\text{A}}$. Comparing (5.3) and (5.2), we obtain the expression for the stress

5.4 $$\sigma_{p}=2\frac{\partial W}{\partial \text{A}}\cdot \text{A}.$$

The same expression for stress is obtained in the GENERIC formalism based on continuum considerations [7] or for specific cases of microscopic bead–spring models [3]. As expected, this is exactly the form of the elastic stress (2.10), with **A** replacing **B**. The second term in (5.3) can be identified as the dissipation

5.5 $$\epsilon_{p}=-\frac{\partial W}{\partial \text{A}}:\overset{▽}{\text{A}}.$$

Combined with $\lambda \overset{▽}{\text{A}}=f(\text{A})$, and an explicit expression for *W*(**A**), this offers a compact expression for the dissipation that is independent of the flow: it depends only on the local value of **A**.

#### (ii) The energy equation

We are now in a position to formulate the energy balance for a polymeric liquid. Multiplying (2.1) by **v**, using (2.2), we obtain

5.6 $$\frac{1}{2}\frac{\partial \rho v^{2}}{\partial t}+\nabla \cdot \left[\left(\frac{\rho v^{2}}{2}+p\right)\text{v}-\eta_{s}\dot{\gamma }\cdot \text{v}-\sigma_{p}\cdot \text{v}\right]=-\epsilon -\frac{1}{2}\sigma_{p}:\dot{\gamma },$$

where

5.7 $$\epsilon =\frac{\eta_{s}}{2}\dot{\gamma }:\dot{\gamma }$$

is the viscous dissipation due to the solvent. Using $\sigma_{p}:\dot{\gamma }/2=\text{d}W/\text{d}t+\epsilon_{p}$, this can be rewritten as

5.8 $$\frac{\text{d}}{\text{d}t}\left(\frac{\rho v^{2}}{2}+W\right)+\nabla \cdot [p\text{v}-\eta_{s}\dot{\gamma }\cdot \text{v}-\sigma_{p}\cdot \text{v}]=-\epsilon -\epsilon_{p},$$

which has the form of a conservation law for the sum of kinetic energy *ρv*^2^/2 and elastic energy *W*. The term in square brackets is the energy flux. The right-hand side represents the dissipation, which has a viscous contribution from the solvent *ϵ*, and a polymeric contribution *ϵ*_*p*_, which according to (5.5) is associated with the relaxation of **A**. The conservation law (5.8) together with the expressions for the stress (5.4) and the dissipation (5.5) are the main results of this section.

Evaluating the elastic stress (5.4) is a repeat of the elastic calculation (2.10) and (2.15). For incompressible solvents, it is sufficient to consider the elastic energy *W*(*I*_1_, *I*_2_), without any dependence on the third invariant.^4^ Then using Jacobi’s formula as well as the Cayley–Hamilton theorem, and that **A** is symmetric, one finds

5.9 $$\frac{\partial W}{\partial \text{A}}=W_{1}\text{I}+W_{2}(\text{tr}(\text{A})\text{I}-\text{A}).$$

Hence (5.4) takes the form

5.10 $$\sigma_{p}=2W_{1}(\text{A}-\text{I})+2W_{2}(\text{I}-\det(\text{A}){\text{A}}^{-1}),$$

which resembles (2.15), but now based on the conformation tensor **A** rather than on the Finger tensor **B**. The relaxation of the conformation tensor gives rise to dissipation *ϵ*_*p*_, which using *W*(*I*_1_, *I*_2_) and (5.5) becomes

5.11 $$\epsilon_{p}=-[W_{1}\text{I}+W_{2}(\text{tr}(\text{A})\text{I}-\text{A})]:\overset{▽}{\text{A}}.$$

It is evident that dissipation vanishes in the absence of relaxation $\overset{▽}{\text{A}}=0$; in that case **A** = **B**, and therefore det(A)=det(B)=1, so that we recover the full structure of elasticity theory.

We note that the dissipation (5.11) can be written in a more elegant form, using the lower convected derivative:^5^

5.12 $$\epsilon_{p}=-W_{1}\text{tr}(\overset{▽}{\text{A}})-W_{2}\text{tr}(\overset{△}{\left(\det(\text{A}){\text{A}}^{-1}\right)}).$$

With this, the full energy balance becomes

5.13 $$\frac{\text{d}}{mt}\left(\frac{\rho v^{2}}{2}+W\right)-\nabla \cdot [\sigma \cdot \text{v}]=-\epsilon +W_{1}\text{tr}(\overset{▽}{\text{A}})+W_{2}\text{tr}(\overset{△}{\left(\det(\text{A}){\text{A}}^{-1}\right)}).$$

This form of the energy equation nicely brings out the symmetry between upper and lower convected derivatives, in relation to **A** and its inverse **A**^−1^. This mirrors the discussion in §3, but now expressed in the context of dissipation.

#### (iii) Non-affine models

So far we have dealt with the physical situation that the constituents follow the flow exactly. As discussed in §3c, using a derivative which is a linear superposition of upper and lower convected derivatives, one can model a situation where the material ‘slips’ relative to the flow. In that case, dissipative processes are described by the relaxation equation in the slipping frame, leading to a form $\lambda {\overset{▽}{\left(\text{A}\right)}}_{a}=f(\text{A})$, where ${\overset{▽}{\left(\text{A}\right)}}_{a}$ is defined in (3.7), which is the upper convected derivative in the polymer frame.

We can follow the same procedure as above, and split d*W*/d*t* into a part that depends on the flow and a part that depends on the relaxation. However, we now invoke ${\overset{▽}{\left(\text{A}\right)}}_{a}$ instead of $\overset{▽}{\text{A}}$ to obtain

5.14 $$\frac{\text{d}W}{\text{d}t}=\frac{\partial W}{\partial \text{A}}:\frac{\text{d}\text{A}}{\text{d}t}=\left(a\frac{\partial W}{\partial \text{A}}\cdot \text{A}\right):\dot{\gamma }+\frac{\partial W}{\partial \text{A}}:{\overset{▽}{\left(\text{A}\right)}}_{a}.$$

We therefore find the stress and dissipation, respectively, as

5.15 $$\sigma_{p}=2a\frac{\partial W}{\partial \text{A}}\cdot \text{A}\;\text{and}\;\epsilon_{p}=-\frac{\partial W}{\partial \text{A}}:{\overset{▽}{\left(\text{A}\right)}}_{a},$$

where **σ**_*p*_ has the same form as (2.10), but with a factor *a* in front of the expression for the stress. This reflects the slip: the polymer is stretched less than expected, making the response ‘softer’ by a fraction *a*. By consequence, the stress can be further expressed as

5.16 $$\sigma_{p}=2aW_{1}(\text{A}-\text{I})+2aW_{2}(\text{I}-\det(\text{A}){\text{A}}^{-1}),$$

while the dissipation reads

5.17 $$\epsilon_{p}=-W_{1}\text{tr}({\left(\overset{▽}{\text{A}}\right)}_{a})-W_{2}\text{tr}(\overset{△}{{\left(\det(\text{A}){\text{A}}^{-1}\right)}_{a}}).$$

For completeness, we again give the energy equation

5.18 $$\frac{\text{d}}{\text{d}t}\left(\frac{\rho v^{2}}{2}+W\right)-\nabla \cdot [\sigma \cdot \text{v}]=-\epsilon +W_{1}\text{tr}({\left(\overset{▽}{\text{A}}\right)}_{a})+W_{2}\text{tr}(\overset{△}{{\left(\det(\text{A}){\text{A}}^{-1}\right)}_{a}}).$$

This is the same as (5.13) but with convected derivatives taken in the slipping frame.

### (c) Rheological models

We conclude by listing a number of frequently considered rheological models, which are usually defined in terms of a constitutive relation for the stress **σ**_*p*_. In the thermodynamic formalism, however, the models are defined by specifying an elastic energy *W*(**A**), complemented by a relaxation equation for **A**. The connection to the polymeric stress as well as the dissipation can be calculated from (5.4) and (5.5) in the affine case and (5.15) in the non-affine case.

(i) **Oldroyd-B/upper convected Maxwell model**
  The upper convected Maxwell model (or: the polymeric part of the Oldroyd-B fluid) is defined by
  5.19 $$\sigma_{p}=\mu (\text{A}-\text{I})\;\text{and}\;\overset{▽}{\text{A}}=-\frac{1}{\lambda }(\text{A}-\text{I}).$$
  One verifies that the elastic energy and dissipation are
  5.20 $$W=\frac{\mu }{2}(\text{tr}(\text{A})-3)=\frac{\mu }{2}(I_{1}-3)\;\text{and}\;\epsilon_{p}=\frac{W}{\lambda }.$$
  Note that *W* is the same as for neo-Hookean solids. Since *W* must be positive (it acquires its minimum for **A** = **I**, where *W* = 0), this implies that *ϵ*_*p*_ ≥ 0, as required. For *λ* → ∞, the dissipation vanishes and one recovers the neo-Hookean solid.
  According to (2.2), the deviatoric stress **τ** is the sum of the solvent and polymer contributions. In the Oldroyd-B model, both can be combined into the single equation
  5.21 $$\tau +\lambda \overset{▽}{\tau }=\eta \dot{\gamma }+\lambda \eta_{s}\overset{▽}{\dot{\gamma }}.$$
  In the limit of vanishing shear rate, (5.21) describes a Newtonian fluid of total viscosity *η* = *η*_*s*_ + *η*_*p*_, the sum of polymeric and solvent contributions. In the limit *λ* → ∞, (5.21) can be integrated to
  5.22 $$\tau =\mu (\text{A}-\text{I})+\eta_{s}\dot{\gamma },$$
  describing a (neo-Hookean) viscoelastic solid.
(ii) **Oldroyd A/lower convected Maxwell model**
  Same as above, but with a relaxation based on the lower convected derivative,
  5.23 $$\sigma_{p}=-\mu (\text{A}-\text{I})\;\text{and}\;\overset{△}{\text{A}}=-\frac{1}{\lambda }(\text{A}-\text{I}).$$
  The elastic energy and dissipation are the same as for the upper convected Maxwell model,
  5.24 $$W=\frac{\mu }{2}(I_{1}-3)\;\text{and}\;\epsilon_{p}=\frac{W}{\lambda }.$$
  While energy *W* is neo-Hookean in terms of $I_{1,\text{A}}=\mathrm{tr}(\text{A})$, the corresponding elastic solid in the limit *λ* → ∞ is not neo-Hookean. Namely, the upper convected derivative gives an elastic limit **A** = **B**^−1^, and the corresponding invariants are related as *I*_1,**A**_ = *I*_2,**B**_.
(iii) **Johnson–Segalman model**
  Same as above, but with a relaxation based on the Gordon–Schowalter derivative
  5.25 $$\sigma_{p}=a\mu (\text{A}-\text{I})\;\text{and}\;{\left(\overset{▽}{\text{A}}\right)}_{a}=-\frac{1}{\lambda }(\text{A}-\text{I}).$$
  The elastic energy and dissipation are the same as for the upper convected Maxwell model,
  5.26 $$W=\frac{\mu }{2}(I_{1}-3)\;\text{and}\;\epsilon_{p}=\frac{\mu }{2\lambda }(I_{1}-3)=\frac{W}{\lambda }.$$
  Note that, owing to the non-affine kinematics of the relaxation law when *a* ≠ 1, the model does not converge to any elastic solid in the limit *λ* → ∞.
(iv) **FENE-P model**
  In the upper/lower convected Maxwell models, both the energy and the dissipation are linear in *I*_1_. However, both *W* and *ϵ*_*p*_ can in general be nonlinear functions of the invariants *I*_1_, *I*_2_. The most popular of such models is the FENE-P model [2,4]. Like other models of the same kind, it is based on the concept of an elastic spring attached to two beads in solution. While a Hookean, non-interacting spring leads to the Oldroyd-B equation, here the spring is nonlinear, so that it cannot be extended beyond a limiting length *L*. This avoids the deficiency of the Oldroyd-B model: that the polymeric stress grows exponentially to infinity in a strong flow (as we already encountered in (4.2)).
  In the nonlinear case, the microscopic model can no longer be solved exactly, so various approximations are used, of which FENE-P is one. The finite extensibility is introduced so that *I*_1_ ≡ tr(**A**) reaches a maximum value *L*^2^, via the stress relation
  5.27 $$\sigma_{p}=\mu f(I_{1})(\text{A}-\text{I}),\;\mathrm{with}\;f(I_{1})=\frac{L^{2}-3}{L^{2}-I_{1}};$$
  clearly, the stress diverges when *I*_1_ = *L*^2^. This stress relation is complemented by a nonlinear relaxation law
  5.28 $$\overset{▽}{\text{A}}=-\frac{1}{\lambda }(f(I_{1})\text{A}-\text{I}).$$
  One verifies that the associated free energy and the dissipation are
  5.29 $$W=\frac{\mu }{2}(L^{2}-3)\ln(f(I_{1}))\;\text{and}\;\epsilon_{p}=\frac{\mu }{2\lambda }f(I_{1})(I_{1}f(I_{1})-3),$$
  and once more *ϵ*_*p*_ ≥ 0. We remark that the FENE-CR model [92] has the same energetic structure as the FENE-P model, but with a slightly different relaxation law, namely $\overset{▽}{\text{A}}=-f(I_{1})(\text{A}-\text{I})/\lambda$.
  Importantly, we note that the elastic energy is the same as that of the Gent model, accounting for finite extensibility in rubber elasticity [93]: the elastic limit *λ* → ∞ of the FENE-P model is the Gent model.
(v) **Giesekus model**
  This is a phenomenological model [2,4] which introduces a term quadratic in **σ**_*p*_ into the equation of motion, which also limits the maximum value of the stress; however, the stress may become arbitrarily large for sufficiently strong flow,
  5.30 $$\sigma_{p}+\lambda {\overset{▽}{\sigma }}_{p}+\alpha \frac{\lambda }{\eta_{p}}\sigma_{p}\cdot \sigma_{p}=\eta_{p}\dot{\gamma }.$$
  This can be written as
  5.31 $$\sigma_{p}=\mu (\text{A}-\text{I})\;\text{and}\;\overset{▽}{\text{A}}=-\frac{1}{\lambda }[\text{A}-\text{I}+\alpha {\left(\text{A}-\text{I}\right)}^{2}],$$
  so that the elastic energy is once more neo-Hookean and the dissipation is
  5.32 $$W=\frac{\mu }{2}(I_{1}-3)\;\text{and}\;\epsilon_{p}=\frac{\mu }{2\lambda }(I_{1}-3)+\frac{\mu \alpha }{2\lambda }(\text{A}:\text{A}-2\text{tr}(\text{A})+3).$$

## 6. Discussion

In summary, we have provided a detailed overview of the relation between the theories of viscoelasticity and of elasticity. In particular, we have explored the ‘elastic correspondence’, by asking which rheological models do (and which do not) converge to an elastic solid, as one considers the limit of infinite relaxation times. The motivation behind this was to highlight universal aspects of soft matter at large deformations, for a broad class of materials. Indeed, the elastic correspondence connects many problems of current interest, such as those shown in figure 1, and offers an original perspective to problems in either fluid or solid mechanics. For example, in §4 we discussed the capillary instability of liquid and solid jets, as shown in figure 1*d*,*e*: the elastic correspondence forms a key element in analysing the break-up of polymeric liquids [48]. Likewise, the fracture of the bridged microemulsion in figure 1*c* is quantitatively similar to the fracture of a purely elastic material [49]. In the same vein, we expect that revisiting classical elastic instabilities such as buckling, wrinkling and creasing from the viscoelastic viewpoint might provide new insight into the dynamical evolution and relaxation of these instabilities. Indeed, experiments have exploited the elastic structure of viscoelastic liquids to characterize surface instabilities in soft materials [58].

By analysing the kinematics of viscoelastic materials in the limit of large relaxation times, we have identified a systematic route to express the energy balance in viscoelastic flows. This is based on the separation of the reversible elastic energy from the dissipation associated with relaxation phenomena. We hope that this will prove useful in the analysis of viscoelastic flows, for example their stability. Indeed, there are many more sources of instability in viscoelastic flows as elastic energy can be stored and transported, to be released elsewhere.

The elastic correspondence relates problems in either fluid or solid mechanics, and thus throws a different light on fluid–structure interactions. Figure 1*b* shows an example of such an interaction, as the liquid drop induces sharp deformations of the (visco)elastic substrate. This article shows how, in principle, the solid can be modelled as a viscoelastic liquid with infinite relaxation time. The general form of the proposed energy equation could help to estimate dissipation, going beyond the usual restrictions of small deformations. From a numerical perspective, the analysis developed here provides a new approach towards computational challenges. For example, the neo-Hookean simulation of §4b for elastic threads has proven to be very efficient, and we have demonstrated how such schemes can also be extended to Newtonian fluids. Conversely, using viscoelastic liquids with infinite relaxation time could offer an attractive, fully Eulerian approach to fluid–structure interaction problems.

## Footnotes

1 More precisely, **B** is a tensor in the vector space defined by **x**, while **C** is a tensor in the vector space defined by **X**. These aspects are made explicit in the curvilinear description given in appendix A.

2 A more precise statement is that the relaxation equation for the conformation tensor is contravariant when the polymer is convected affinely with the flow.

3 We define the reference state at *t* = 0, which implies that **B**(0) = **I**. A stress-free initial condition implies **A**(0) = **I**, which is the case in (3.13).

4 We remark that in general *I*_3_ ≠ 1 for incompressible flows. However, its effect on **σ**_*p*_ is that it adds an isotropic contribution proportional to *W*_3_ = ∂*W*/∂*I*_3_, which can be absorbed in the pressure. Namely, given that $\sigma_{p}=2\frac{\partial W}{\partial \text{A}}\cdot \text{A}$, the contribution of *I*_3_ to the stress tensor is $2W_{3}\frac{\partial I_{3}}{\partial \text{A}}\cdot \text{A}$. Owing to Jacobi’s formula, $\frac{\partial I_{3}}{\partial \text{A}}=\det(\text{A}){\text{A}}^{-1}$, so that the corresponding contribution becomes $2W_{3}\det(\text{A})\text{I}$, the effect of *I*_3_ on the stress is indeed isotropic. Similarly, one can demonstrate that, for incompressible flows, the contribution of *W*_3_ to *ϵ*_*p*_ is a total derivative d/d*t*, and is therefore not ‘dissipative’ and can thus be absorbed by a redefinition of *W*. Hence, it suffices to consider *W*(*I*_1_, *I*_2_). For compressible flows, *W*_3_ must indeed be included explicitly [91].

5 Here we made use of the identity

$$\text{tr}(\text{A})\text{I}:\overset{▽}{\text{A}}-\text{A}:\overset{▽}{\text{A}}=\text{tr}(\text{A})\text{tr}(\overset{▽}{\text{A}})-\text{A}:\overset{▽}{\text{A}}=\text{tr}(\overset{△}{\left(\det(\text{A}){\text{A}}^{-1}\right)}),$$

which can be verified directly upon invoking the Cayley–Hamilton theorem (2.14).

## Data accessibility

This article has no additional data.

## Appendix A. Curvilinear formulation of viscoelasticity

**(a) Kinematics of deformation**

The purpose of this appendix is to rephrase the results of the main text in terms of curvilinear coordinates. This allows for a rigorous analysis of the physical assumptions underlying the equation of motion for the conformation tensor, **A**. For a detailed description of the kinematics discussed below, the reader can refer to the book by Green & Zerna [94].

We define curvilinear coordinates *q*^*i*^, which are material points that move along affinely with the flow, as specified more precisely in (11). The current position vector is defined as **x**(*q*^*i*^, *t*), while the position of the reference (or initial) configuration reads **X**(*q*^*i*^) = **x**(*q*^*i*^, *t* = 0). The latter is independent of time. The distance d*s* between two neighbouring points *q*^*i*^ and *q*^*i*^ + *dq*^*i*^ reads

A 1 $$\text{d}s^{2}=\left(\frac{\partial \text{x}}{\partial q^{i}}\cdot \frac{\partial \text{x}}{\partial q^{j}}\right)\text{d}q^{i}\;\text{d}q^{j}=g_{ij}\;\text{d}q^{i}\text{d}q^{j},$$

where *g*_*ij*_ is the current metric tensor. Similarly, the reference distance d*S* follows as

A 2 $$\text{d}S^{2}=\left(\frac{\partial \text{X}}{\partial q^{i}}\cdot \frac{\partial \text{X}}{\partial q^{j}}\right)\text{d}q^{i}\text{d}q^{j}=G_{ij}\;\text{d}q^{i}\text{d}q^{j},$$

where *G*_*ij*_ is the reference metric. Stretching of material elements follows from changes in length

A 3 $$\text{d}s^{2}-\text{d}S^{2}=(g_{ij}-G_{ij})\text{d}q^{i}\text{d}q^{j},$$

so strain is encoded in the difference between the current and reference metric.

We now construct the current vector space, using the covariant and contravariant basis vectors

A 4 $${\text{e}}_{i}=\frac{\partial \text{x}}{\partial q^{i}}\;\text{and}\;{\text{e}}^{j}=\frac{\partial q^{j}}{\partial \text{x}},$$

derived from the current position **x**. The covariant basis vectors **e**_*i*_ are local tangents to the material lines in the deformed configuration. The contravariant vectors **e**^*i*^ form a reciprocal basis, owing to the property ${\text{e}}_{i}\cdot {\text{e}}^{j}=\text{d}q^{j}/\text{d}q^{i}=\delta_{i}^{j}$. Using this basis, we can define the metrics

A 5 $$g_{ij}={\text{e}}_{i}\cdot {\text{e}}_{j},\;g^{ij}={\text{e}}^{i}\cdot {\text{e}}^{j},$$

so that *g*_*ij*_ can be used to lower indices, while the inverse *g*^*ij*^ raises indices. Similarly, one can construct the reference vector space, using ‘reference’ basis vectors

A 6 $${\text{E}}_{i}=\frac{\partial \text{X}}{\partial q^{i}}\;\text{and}\;{\text{E}}^{j}=\frac{\partial q^{j}}{\partial \text{X}}.$$

The associated metric for this basis as well as its inverse are defined by

A 7 $$G_{ij}={\text{E}}_{i}\cdot {\text{E}}_{j},\;G^{ij}={\text{E}}^{i}\cdot {\text{E}}^{j};$$

the **E**_*i*_ are local tangents to the material lines in the reference configuration.

We now wish to express the metrics in terms of the mapping **F** = ∂**x**/∂**X**. In particular, we wish to show that Green’s deformation tensor **C** = **F**^T^ · **F** and the Finger tensor **B** = **F** · **F**^T^ can be written as

A 8 $$\text{C}=g_{ij}{\text{E}}^{i}⊗{\text{E}}^{j},\;\text{B}=G^{ij}{\text{e}}_{i}⊗{\text{e}}_{j}.$$

To demonstrate this, we write (1) as

A 9 $$\text{d}s^{2}={\left(\frac{\partial \text{x}}{\partial \text{X}}\cdot \frac{\partial \text{X}}{\partial q^{i}}\right)}^{\text{T}}\cdot \left(\frac{\partial \text{x}}{\partial \text{X}}\cdot \frac{\partial \text{X}}{\partial q^{j}}\right)\text{d}q^{i}\text{d}q^{j}={\text{E}}_{i}\cdot ({\text{F}}^{\text{T}}\cdot \text{F})\cdot {\text{E}}_{j}\;\text{d}q^{i}\text{d}q^{j}.$$

Comparing with (1), we indeed see that *g*_*ij*_ are the covariant components of **C** = **F**^T^ · **F** when expressed using the basis **E**^*i*^. Hence, we obtain the first identity in (8). It is important to keep track of the basis used to express the tensor [95]; for example, pairing *g*_*ij*_ with the basis **e**^*i*^, one recovers the identity tensor, **I** = *g*_*ij*_**e**^*i*^ ⊗ **e**^*j*^.

Similarly, we rewrite (2) as

A 10 $$\text{d}S^{2}={\left(\frac{\partial \text{X}}{\partial \text{x}}\cdot \frac{\partial \text{x}}{\partial q^{i}}\right)}^{\text{T}}\cdot \left(\frac{\partial \text{X}}{\partial \text{x}}\cdot \frac{\partial \text{x}}{\partial q^{j}}\right)\text{d}q^{i}\text{d}q^{j}={\text{e}}_{i}\cdot ({\text{F}}^{-T}\cdot {\text{F}}^{-1})\cdot {\text{e}}_{j}\;\text{d}q^{i}\text{d}q^{j}.$$

Now we see that *G*_*ij*_ are the covariant components of ${\text{B}}^{-1}={\text{F}}^{-T}\cdot {\text{F}}^{-1}$ when expressed using the basis **e**^*i*^. Since the inverse of the reference metric is defined as $G_{ik}G^{kj}=\delta_{i}^{j}$, we obtain the second identity in (8). Again, pairing *G*_*ij*_ with the basis **E**^*i*^, one recovers the identity tensor, **I** = *G*_*ij*_**E**^*i*^ ⊗ **E**^*j*^.

**(b) Flow**

Now, we investigate the effect of flow on the metric. First, we define the velocity as

A 11 $$\text{v}={\left(\frac{\text{d}\text{x}}{\text{d}t}\right)}_{q^{i}}=v^{i}{\text{e}}_{i},$$

expressed using the basis defined by (4), where from now on d/d*t* means the time derivative at constant material points *q*^*i*^. Using (5) and (4), the time derivative of the metric tensor is

A 12 $$\begin{array}{rl} \frac{\text{d}g_{ij}}{\text{d}t} & =\left(\frac{\partial (v^{k}{\text{e}}_{k})}{\partial q^{i}}\cdot {\text{e}}_{j}\right)+\left({\text{e}}_{i}\cdot \frac{\partial (v^{m}{\text{e}}_{m})}{\partial q^{j}}\right)\equiv (v_{;i}^{k}{\text{e}}_{k})\cdot {\text{e}}_{j}+{\text{e}}_{i}\cdot ({\text{e}}_{m})v_{;j}^{m}\\ & =g_{kj}v_{;j}^{k}+g_{mi}v_{;j}^{m}=v_{j;i}+v_{i;j}\equiv {\dot{\gamma }}_{ij}, \end{array}$$

where we used the definition of the covariant derivative, denoted by (..)_;*j*_. Hence, the rate of strain tensor $\dot{\gamma }$ directly gives the change of the metric of material coordinates by the flow. Remembering that *B*^*ij*^ = *G*^*ij*^ (cf. (8)), we see that the Finger tensor evolves according to

A 13 $$\frac{\text{d}B^{ij}}{\text{d}t}=\frac{\text{d}}{\text{d}t}\left(\frac{\partial q^{i}}{\partial \text{X}}\cdot \frac{\partial q^{j}}{\partial \text{X}}\right)=0$$

during flow. This time derivative vanishes because the reference state **X**(*q*^*i*^) is independent of time. Note, however, that the covariant components *B*_*ij*_ are not constant in time, since

A 14 $$\frac{\text{d}B_{ij}}{\text{d}t}=\frac{\text{d}}{\text{d}t}(g_{ik}g_{jm}B^{km})=2{\dot{\gamma }}_{ik}B_{j}^{k}.$$

For a general tensor **A**, the derivatives d*A*^*ij*^/d*t* and d*A*_*ij*_/d*t*, respectively, correspond to the components of the upper and lower convected derivatives [61,96], i.e.

A 15 $$\overset{▽}{\text{A}}=\frac{\text{d}A^{ij}}{\text{d}t}{\text{e}}_{i}⊗{\text{e}}_{j},\;\overset{△}{\text{A}}=\frac{\text{d}A_{ij}}{\text{d}t}{\text{e}}^{i}⊗{\text{e}}^{j}.$$

The equivalence with the definitions (2.17) and (2.18) follows from transforming *A*^*ij*^ and *A*_*ij*_, respectively, from the Lagrangian (co-moving) material coordinates *q*^*i*^ to an Eulerian coordinate system ${\bar{q}}^{i}$ that is fixed in space. In this fixed coordinate system, the tensor components denoted by ${\bar{A}}^{ij}$ can be obtained using the transformation $F_{i}^{k}=\partial {\bar{q}}^{k}/\partial q^{i}$. Transforming d*A*^*ij*^/d*t* to the fixed frame then gives

A 16 $$F_{i}^{p}\left(\frac{\text{d}A^{ij}}{\text{d}t}\right)F_{j}^{q}=F_{i}^{p}\frac{\text{d}}{\text{d}t}[{\left(F^{-1}\right)}_{k}^{i}{\bar{A}}^{km}{\left(F^{-1}\right)}_{m}^{j}]F_{j}^{q}.$$

On the right-hand side, we recognize the definition (2.17) for $\overset{▽}{\text{A}}$, now in the form of the components of the fixed Eulerian system ${\bar{q}}^{i}$. In a similar fashion, one derives (15) for the lower convected derivative. Hence, d*B*^*ij*^/d*t* = 0 implies that the upper convected derivative of the Finger tensor vanishes, i.e. $\overset{▽}{\text{B}}=0$.

**(c) Elasticity**

In the theory of elasticity, the energy density *W* is a function of the invariants of **B**. Assuming incompressibility $I_{3}=\det(\text{B})=1$, the energy is of the form *W*(*I*_1_, *I*_2_), where the first and second invariants are defined as

A 17 $$I_{1}=B_{i}^{i}=g_{ij}B^{ij},\;I_{2}=\frac{1}{2}({\left[g_{ij}B^{ij}\right]}^{2}-B_{ij}B^{ij})=\frac{1}{2}({\left[g_{ij}B^{ij}\right]}^{2}-g_{im}g_{jn}B^{mn}B^{ij}).$$

The expression for stress is obtained from time derivatives, according to the virtual work principle

A 18 $$\frac{1}{2}\sigma^{ij}{\dot{\gamma }}_{ij}=\frac{\text{d}W}{\text{d}t}=W_{1}\frac{\text{d}I_{1}}{\text{d}t}+W_{2}\frac{\text{d}I_{2}}{\text{d}t}.$$

Using d*B*^*ij*^/d*t* = 0, we find

A 19 $$\frac{\text{d}I_{1}}{\text{d}t}=\frac{\text{d}g_{ij}}{\text{d}t}B^{ij}={\dot{\gamma }}_{ij}B^{ij},\;\frac{\text{d}I_{2}}{\text{d}t}=\frac{\text{d}g_{ij}}{\text{d}t}\frac{\partial I_{2}}{\partial g_{ij}}={\dot{\gamma }}_{ij}(I_{1}B^{ij}-B_{n}^{i}B^{nj}),$$

both of which are proportional to ${\dot{\gamma }}_{ij}$. Hence, from (18) we can read off the stress as

A 20 $$\sigma^{ij}=2\frac{\partial W}{\partial g_{ij}}=2W_{1}B^{ij}+2W_{2}(I_{1}B^{ij}-B_{n}^{i}B^{nj}).$$

**(d) Viscoelasticity**

When an elastic *liquid* is suddenly arrested, the polymer will relax towards an isotropic equilibrium confirmation. The system slowly forgets about the history of deformation prior to the arrest, and all stress and polymer stretches are ultimately relaxed. When expressing the polymer deformation in terms of the elementary lengths between *q*^*i*^ and *q*^*i*^ + d*q*^*i*^, we can still write

A 21 $$\text{d}s^{2}-\text{d}S^{2}=(g_{ij}-G_{ij})\text{d}q^{i}\text{d}q^{j},$$

and we associate an elastic energy with the polymer strain. Owing to the fading memory of the initial state **x**(*q*^*i*^, *t* = 0), however, the object *G*_*ij*_ can no longer be identified with the time-independent metric of this initial condition. Instead, *G*_*ij*_ reflects the metric of the ‘instantaneous reference state’ **X**(*q*^*i*^, *t*) that progressively tends to evolve towards the current state.

From the above, we have a very clear definition of flow and relaxation: at fixed material point *q*^*i*^, flow refers to the time dependence of **x**, while relaxation implies the time dependence of **X**. We now exploit this further using (7) and (6), and obtain

A 22 $$G_{ij}=\frac{\partial \text{X}}{\partial q^{i}}\cdot \frac{\partial \text{X}}{\partial q^{i}},\;G^{ij}=\frac{\partial q^{i}}{\partial \text{X}}\cdot \frac{\partial q^{j}}{\partial \text{X}}.$$

We can then define the conformation tensor **A**, whose eigenvalues give the stretches of the polymer. This is in direct analogy to the Finger tensor **B**, the only difference being that the stretches need to be measured with respect to the instantaneous reference state **X**(*q*^*i*^, *t*), rather than **x**(*q*^*i*^, *t* = 0). Hence,

A 23 $$\text{A}=\left(\frac{\partial q^{i}}{\partial \text{X}}\cdot \frac{\partial q^{j}}{\partial \text{X}}\right){\text{e}}_{i}⊗{\text{e}}_{j}=G^{ij}{\text{e}}_{i}⊗{\text{e}}_{j}.$$

At the start of §5, we referred to $\text{X}(q^{i},t)={\text{X}}^{*}$, so that we there defined $\text{A}={\text{F}}^{*}\cdot {\text{F}}^{*T}$. The components *A*^*ij*^ in (23) do not involve any flow, but only relaxation, as relaxation includes time-dependent effects only of **X**(*q*^*i*^, *t*). Hence, if we wish to express a relaxation law directly in terms of the conformation tensor **A**, we necessarily arrive at

A 24 $$\frac{\text{d}A^{ij}}{\text{d}t}=\frac{1}{\lambda }f^{ij}(\text{A}),$$

which in the fixed frame corresponds to the upper convected derivative. By contrast, the covariant components *A*_*ij*_ = *g*_*ik*_*g*_*jm*_ *A*^*km*^ do exhibit a time dependence due to the flow, via d*g*_*ij*_/d*t*. Hence, to quantify the relaxation in terms of the conformation tensor **A**, one automatically singles out the upper convected derivatives as the appropriate time derivative.

In analogy with elasticity theory, we introduce an elastic energy *W*(*I*_1_, *I*_2_) associated with the first and second invariants of **A**. Once again we employ the virtual work principle, but now including dissipation *ϵ*_*p*_,

A 25 $$\frac{1}{2}\sigma^{ij}{\dot{\gamma }}_{ij}=\frac{\text{d}W}{\text{d}t}+\epsilon_{p}.$$

The dissipation is necessary since the elastic energy exhibits an extra time dependence associated with relaxation,

A 26 $$\frac{\text{d}W}{\text{d}t}=\frac{\partial W}{\partial g_{ij}}{\dot{\gamma }}_{ij}+\frac{\partial W}{\partial A^{ij}}\frac{\text{d}A^{ij}}{\text{d}t}.$$

Again, the terms proportional to ${\dot{\gamma }}_{ij}$ provide the stress, so that stress and dissipation can be separated as

A 27 $$\sigma^{ij}=2\frac{\partial W}{\partial g_{ij}},\;\epsilon_{p}=-\frac{\partial W}{\partial A^{ij}}\frac{\text{d}A^{ij}}{\text{d}t}.$$

## Appendix B. Integrating the upper convected Maxwell model

In order to integrate the upper convected Maxwell model, we write (2.7) using (2.17) as

B 1 $$\text{F}\cdot \left[\frac{\text{d}}{\text{d}t}({\text{F}}^{-1}\cdot \text{A}\cdot {\text{F}}^{-T})\right]\cdot {\text{F}}^{\text{T}}=-\frac{1}{\lambda }(\text{A}-\text{I}),$$

so that, upon defining $\text{D}={\text{F}}^{-1}\cdot \text{A}\cdot {\text{F}}^{-T}$, we can write

B 2 $$\frac{\text{d}\text{D}}{\text{d}t}=-\frac{1}{\lambda }(\text{D}-{\text{F}}^{-1}\cdot {\text{F}}^{-T}).$$

Note that **A** is defined on the Eulerian domain, while **D** is defined on the reference domain. Therefore, its time derivative does not contain any convective terms, and one can treat (B 2) as an ordinary differential equation. The term $\text{g}(t)={\text{F}}^{-1}(t)\cdot {\text{F}}^{-T}(t)$ can be treated as some function of time that is imposed externally by the flow. One verifies that the solution to (B 2) reads

B 3 $$\text{D}(t)=e^{-t/\lambda }{\text{D}}_{0}+\frac{1}{\lambda }\int_{0}^{t}\;\text{d}t^{\prime }\;{\text{e}}^{-\left(t-t^{\prime }\right)/\lambda }\text{g}(t^{\prime }),$$

where **D**_0_ = **D**(0) is a constant of integration. Transforming the result back to the Eulerian domain, by the operation **F**(*t*) · ( · · · ) · **F**^T^(*t*), we obtain

B 4 $$\text{A}(t)={\text{e}}^{-(t/\lambda )}\text{F}(t)\cdot {\text{D}}_{0}\cdot {\text{F}}^{\text{T}}(t)+\frac{1}{\lambda }\int_{0}^{t}\;\text{d}t^{\prime }\;{\text{e}}^{-\left(t-t^{\prime }\right)/\lambda }\text{F}(t)\cdot \text{g}(t^{\prime })\cdot {\text{F}}^{\text{T}}(t).$$

For a stress-free initial condition, **D**_0_ = **I**, this gives (3.13).
